# Expert Algorithm for Substance Identification Using Mass Spectrometry: Statistical Foundations in Unimolecular Reaction Rate Theory

**DOI:** 10.1021/jasms.3c00089

**Published:** 2023-05-31

**Authors:** Glen P. Jackson, Samantha A. Mehnert, J. Tyler Davidson, Brandon D. Lowe, Emily A. Ruiz, Jacob R. King

**Affiliations:** † Department of Forensic and Investigative Science, West Virginia University, Morgantown, West Virginia 26506, United States; ‡ C. Eugene Bennett Department of Chemistry, West Virginia University, Morgantown, West Virginia 26506, United States

**Keywords:** spectral comparisons, spectral algorithm, search algorithm, forensic science, compound identification, binary classification, drug identification

## Abstract

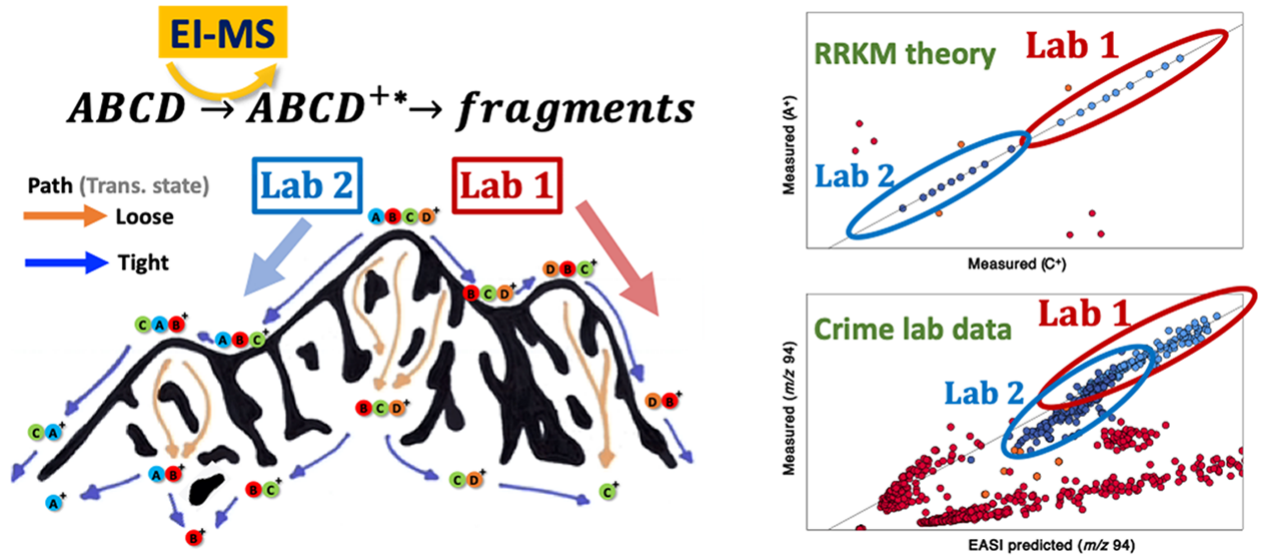

This study aims to resolve one of the longest-standing problems in mass spectrometry, which is how to accurately identify an organic substance from its mass spectrum when a spectrum of the suspected substance has not been analyzed contemporaneously on the same instrument. Part one of this two-part report describes how Rice–Ramsperger–Kassel–Marcus (RRKM) theory predicts that many branching ratios in replicate electron–ionization mass spectra will provide approximately linear correlations when analysis conditions change within or between instruments. Here, proof-of-concept general linear modeling is based on the 20 most abundant fragments in a database of 128 training spectra of cocaine collected over 6 months in an operational crime laboratory. The statistical validity of the approach is confirmed through both analysis of variance (ANOVA) of the regression models and assessment of the distributions of the residuals of the models. General linear modeling models typically explain more than 90% of the variance in normalized abundances. When the linear models from the training set are applied to 175 additional known positive cocaine spectra from more than 20 different laboratories, the linear models enabled ion abundances to be predicted with an accuracy of <2% relative to the base peak, even though the measured abundances vary by more than 30%. The same models were also applied to 716 known negative spectra, including the diastereomers of cocaine: allococaine, pseudococaine, and pseudoallococaine, and the residual errors were larger for the known negatives than for known positives. The second part of the manuscript describes how general linear regression modeling can serve as the basis for binary classification and reliable identification of cocaine from its diastereomers and all other known negatives.

## Introduction

Since its first demonstration in the late 1930s,^1^ electron ionization (née impact) mass spectrometry (EI-MS) has proven to be a powerful tool for identifying organic substances.^2−4^ More than 80 years since its introduction, EI-MS in the form of gas chromatography–mass spectrometry (GC-MS) continues to be one of the most commonly employed methods of compound identification in forensic science,^5,6^ metabolomics,^7,8^ flavor and fragrance,^5^ toxicology,^9^ and pharmacology.^10^ Given that there are now more than 300,000 compounds in the latest EI-MS database from NIST, computerized approaches to compound identification have long been a necessity.^11−16^

Ignoring the importance of GC retention times for a moment, the most common approach to identifying a substance from its mass spectrum is by performing a library search, using either the apex spectrum of a GC peak or the average spectrum across a GC peak. Library search algorithms then compare the presence and abundance of peaks in the questioned mass spectrum (the unknown) to those in known exemplar spectra of reference samples in a database. Depending on the database vendor and the algorithm employed, the software typically returns a list of the top 10 closest matches with some measure of spectral similarity assigned to each pairwise comparison between the questioned spectrum and the reference spectra.^17,18^

Once a best candidate is chosen, an analyst, such as a seized drug analyst, is usually required to manually compare the unknown and reference spectra. Organizations such as the World Anti-Doping Agency (WADA), United Nations Office on Drugs and Crime (UNODC), and the American Society for Testing and Materials (ASTM) all provide acceptance criteria that analysts are expected to use when evaluating the peak abundances of a questioned sample relative to those of a reference spectrum.^19−25^ These acceptance criteria generally permit the abundances of peaks in the questioned spectrum to fall within ±20% of their respective abundances in the reference spectrum,^19−25^ although agencies vary in their stringencies.^26,27^ These acceptance criteria are generally in agreement with typical uncertainties observed in replicate spectra of standards collected in crime laboratories.^28^

The use of spectral libraries is complicated by the fact that spectra derive from various types of mass analyzers, and the spectra vary in their quality, the presence of background ions, and the occurrence of mass bias.^29^ Furthermore, instruments have different geometries, operating conditions, and other idiosyncrasies that contribute to the overall variance in ion abundances of replicate spectra.^15,28,30^ The choice of tuning algorithm and tuning frequency also plays a role in the variability of replicate spectra.^31^ These differences within and between instruments have deleteriously affected the ability of algorithms to rank or identify substances correctly.^12,13,15,17,18,32−36^ All of these factors negatively affect the ability of current algorithms to make true positive identifications, even when a reference spectrum of the questioned substance is present in the database. For example, most algorithms typically only provide around 80% accuracy in ranking the correct identity in the #1 position.^13,37−44^ For reasons that will become apparent later, the inclusion of replicate spectra of each substance can significantly improve identification rates.^12,45,46^

Various methods have been developed to improve the confidence in substance identification using spectral comparison techniques, such as changing the weighting factors,^40,41,43,44,47^ changing the peak selection or abundance-normalization method,^37,48^ modifying the results based on experimental information after the spectrum has already been collected,^49^ and increasing the size of the library.^12^ Other improvements include the use of partial and semipartial correlations^50^ and wavelet and Fourier transformations to increase the accuracy of the identification algorithms.^51^

Given the breadth of potential applications and the varying weight of false positives and false negatives in different applications, there is no consensus as to which approach, or algorithm, is “best”. Therefore, analysts must select an approach that is simply the best fit for their purpose.^10,12,18^ As indicated earlier, the most common approach to improving the success rate of mass spectral identifications is to combine the mass spectral information with independent information, such as the retention time or retention index.^7,8,40,49,52−59^ However, if the unknown material has not or cannot be analyzed on the same instrument, the database retention times/indices may not be sufficiently reliable to enable the differentiation of structurally similar compounds. In cases involving coelution, chromatographic peaks can be deconvoluted from each other and/or from background ions and thereby increase the success of mass spectral identifications.^49,50,55,60−62^

## The Random and Nonrandom Variance of Replicate Spectra

One aspect of spectral comparison algorithms that is often overlooked is the assumption, and oftentimes mathematical requirement, that any variance in the *relative* abundance of peaks within a replicate spectrum is randomly or independently variable at each *m*/*z* value.^48,63^ Such a mathematical requirement has been assumed since the first use of computational approaches to background-subtraction^64^ or spectral deconvolution into discrete component spectra,^60,65,66^ whether using simultaneous linear equations^65^ or matrix theory.^67,68^ By default, deconvolution algorithms explicitly assume unit correlation among the *absolute* signals of fragments as a function of time, or scan number, and they implicitly assume that any unexplained variance in a given scan at a specific *m*/*z* value is random.^49,50,55,60,61,65,67−70^ Furthermore, to have statistical validity, most measures of spectral similarity and dissimilarity between questioned and reference spectra also require independent variance, i.e., no correlation, in the *relative* abundance at each *m*/*z* value within replicate spectra.^15,37,71^ As an example of this reliance, a recent and extremely effective approach to spectral comparisons uses combined unequal variance *t*-tests at each *m*/*z* value to compare questioned and known spectra. Combining the results of independent *t*-tests explicitly requires independent variability of each *t*-test to enable the computation of random match probabilities.^72−74^ However, as indicated elsewhere,^28^ and as we will show below, replicate spectra still contain strong correlations in the normalized abundances of peaks, so the different *m*/*z* values are not independently variable. Supplemental Figure S1 supports this contention by showing obvious correlations and anticorrelations in the normalized abundances of certain fragments of cocaine across a chromatographic peak.

## The Value of Cross-Correlations in Replicate Spectra

In the 1960s, Crawford and Morrison noted that systematic differences in fragmentation patterns occur when a mass spectrum of a substance was collected by sweeping the magnetic field instead of the ion acceleration voltage of a magnetic sector instrument.^75^ In 1979, van Marlen et al. also noted that the abundances of peaks in replicate spectra were not independently variable and that when spectra deviate from a reference spectrum, it was “virtually impossible to take the correlations between the errors for the different *m/e* (sic) values into account”.^48^ Here, an “error” is the deviate abundance at one *m*/*z* value between a questioned peak abundance and a reference peak abundance.

The difficulty in accounting for correlations between residuals has caused almost all search algorithms since then to assume that peak abundances in replicate spectra are independently variable,^76^ including the traditional and popular peptide-scoring algorithms based on tandem mass spectra of protonated precursors.^36,77−81^ However, by considering correlations between theoretical and measured fragment ions in tandem mass spectra of peptides, Fu et al. were able to reduce the peptide mismatch rate from their tandem mass spectra by as much as 10%.^82^ Of course, such approaches are only possible when the sequence is known for a precursor ion and the theoretical fragment ions can be accurately predicted. Driver et al. also used covariance analysis between fragments in replicate spectra to improve the selectivity of their peptide identification algorithm, and they also demonstrated the ability to identify mechanistic relationships between fragment-fragment pairs, such as complementary b/y fragments or consecutive fragments, like the loss of CO from a b ion to form a corresponding a ion. Related to the present study, their covariance mapping was conducted on simple replicate spectra that were collected without deliberate variance in the magnitude or mechanism of perturbation.^83−86^ Zhang’s group showed that partial and semipartial correlations could reduce the false discovery rate of library searches of EI spectra by a few percent.^50^ Therefore, the ability to incorporate cross-correlations of replicate spectra into mass spectral comparison algorithms is a demonstrated mechanism to improve their performance.

Mass bias and spectral tilting can be thought of as *m*/*z*-dependent correlations in which low mass ions correlate with one another, high mass ions correlate with one another, but low mass and high mass ions anticorrelate with one another. McLafferty’s group and Dromey have both shown that by incorporating correction terms, or scaling terms, to account for mass bias in replicate spectra, the unexplained variance in replicate peak abundances can be reduced by as much as 40%, and the reliability of search algorithms can be increased by as much as 10%.^11,87,88^ As shown below, our expert algorithm for substance identification (EASI) naturally accounts for correlations between peaks that derive from mass bias and spectral tilting.

## Multivariate Methods

By design, multivariate methods of spectral comparisons tend to account for the covariance or correlations between ion abundances in replicate spectra. Sigman and Clark examined the two-dimensional cross-correlations in replicate spectra of high explosives,^89^ but the cross-correlations were examined as a function of a deliberate perturbation, i.e., differing collision energies, and the cross-correlations were not used to support compound identification, unlike the present work. In mass spectrometry applications, principal component analysis (PCA) has been used in two main ways: (1) to resolve or deconvolute mass spectra of mixtures,^66,68,70,90^ and (2) to relate a spectrum to other classes or structures within a database.^74,91−96^ The latter approach has also been used in conjunction with discriminant analysis and binary classification algorithms to enable the classification of spectra to known identities.^97−107^ Finally, machine learning and artificial intelligence methods have existed since the early 1970s,^108^ and they continue to be explored as methods to both identify known compounds in a library and to propose structures for compounds that are not in a library.^47,57,78,79,109−115^ Whereas the predictive power of sophisticated computational techniques is likely to continually advance, very few of the articles described so far tackle the difficult problem of discriminating between spectrally similar compounds collected on different instruments and without reference spectra from those instruments.

The ability to resolve spectrally and structurally similar compounds, like isomers, using their EI-MS spectra has traditionally relied on the knowledge that certain ions are spectrally more unique or more valuable for discriminating isomers than others. For example, strongly correlating ions are thought to provide near-constant ratios of abundances that can be used to help discriminate between structurally similar compounds like positional isomers^94,99^ and cocaine diastereomers.^116−118^ Indeed, in the 1960s Tal’roze and Raznikov showed that if the correct ion ratios are taken into account, just three or four pairs of ratios are enough to resolve hundreds of compounds successfully.^119,120^ In the case of cocaine, although the GC retention times can usually resolve the four major diastereomers of allococaine, cocaine, pseudococaine, and pseudoallococaine, mass spectral differentiation of the diastereomers within an instrument has been proposed through the ratios of peak abundances at *m*/*z* 94:96 and *m*/*z* 152:150.^116−118^ However, the tactic of finding and examining specific pairs of ions to differentiate isomers of drugs^94,99^ is a time-consuming approach that is not readily scalable to all isomers of all drugs or compounds. Analysts need an objective algorithm that is sufficiently flexible in identifying the ion ratios or correlations that are most characteristic or most discriminating for each substance without human intervention.

## Expert Algorithm for Substance Identification

We propose a new paradigm for mass spectral identification that does not require spectral similarity between the questioned spectrum and any of the reference spectra of the same substance to make a reliable identification. The described approach follows several guiding philosophies of valid computational methods,^78^ including that (1) it be rooted in a solid scientific/mathematical basis, (2) it should have as few user-definable parameters as possible, (3) if possible, parameters should be learned from the data, and (4) if user-definable parameters are unavoidable, there should be very clear instructions on how to set these parameters depending on the experimental setup. As we will show, EASI can correctly discriminate known positives and known negatives even when known negatives are more similar to an exemplar or consensus spectrum^121^ than other known positives, and EASI outperforms other algorithms even when data are collected on different types of mass analyzers. In this first demonstration, we show that EASI can reliably resolve a drug like cocaine from its diastereomers, even in the absence of chromatographic information.

The foundational basis and proof-of-concept of EASI is described in two parts. Part 1, here, describes how the kinetics of unimolecular fragmentation can be empirically modeled in a retrospective and passive manner from any existing database of replicate spectra of a reference material. Such replicates already exist in most crime laboratory settings because most jurisdictions already require spectra of standards to be collected contemporaneously with casework samples. Part 1 also describes how to assess and use the correlated variance in the replicates to build empirical models with which to compare the measured values. In part 2, the spectral similarity between modeled ion abundances and measured ion abundances is assessed in a variety of ways to enable binary classification using receiver operating characteristic (ROC) curves^13,34,122,123^ to discriminate between cocaine and its diastereomers, selectively and confidently, and even when the spectra were collected decades apart and on a variety of different types of mass spectrometers. Because almost all the questioned spectra of cocaine can be resolved from those of its diastereomers, which are the most likely to cause false positives, we assume the questioned cocaine spectra can easily be discriminated from all other known negatives. Such verification and validation is easily supported by conventional algorithms.

## Materials and Methods

A drug standard mixture analyzed by Laboratory 1 contained methamphetamine (1500 ppm), cocaine (1500 ppm), and hydromorphone (2000 ppm). Methamphetamine and hydromorphone were supplied by Sigma-Aldrich (St. Louis, MO, USA), cocaine was supplied by Mallinckrodt (St. Louis, MO, USA), and the methanol solvent was supplied by Alfa Aesar (Haverhill, MA, USA). The drug standard mixture analyzed by Laboratory 2 was comprised of ecgonine methyl ester (5050 ppm), cocaine (5050 ppm), 6-monoacetylmorphine (6-MAM) (5700 ppm), diacetylmorphine (DAM) (5700 ppm), and fentanyl (5200 ppm). The ecgonine methyl ester and cocaine were supplied by Sigma-Aldrich, the 6-MAM, DAM, and fentanyl were supplied by Lipomed (Cambridge, MA, USA), and the methanol solvent was supplied by Fisher Scientific (Waltham, MA, USA). The training set and test set are composed of data from abundant chromatographic peaks, so they represent somewhat ideal conditions that will not always be realized in casework. Future work, currently in progress, will consider the influence of absolute signal intensity on the performance of EASI.

An Agilent Technologies 7890 GC-5977 MS with a 12 m × 200 μm × 0.33 μm HP-5 (5% phenyl-methylpolysiloxane) column (Agilent J&W Columns, Santa Clara, CA, USA) was used by Laboratory 1. The GC method involved a 1 μL injection volume into a 220 °C injection port and a 100:1 split ratio. The initial oven temperature was held at 80 °C for 1.5 min, before being ramped to 270 °C at 50 °C/min and then held for 1.67 min. The method also included a second ramp to 290 °C with a 35 °C/min ramp rate and a 2.7 min hold. Helium was used as the carrier gas with a 1 mL/min flow rate. The transfer line temperature was set to 290 °C. The mass spectrometer scan range was *m*/*z* 30–650, with a 0.80 min solvent delay and a scan rate of 2852 Da/sec. The EI source temperature was 230 °C and the quadrupole temperature was 150 °C.

Laboratory 2 also used an Agilent Technologies 7890 GC-5977 MS; however, Lab 2 used a 30 m × 250 μm × 0.25 μm DB-5MS (5% phenyl-methylpolysiloxane) column (Agilent J&W Columns). The GC-MS method included a 0.2 μL injection volume into a 280 °C injection port and a 20:1 split ratio. The initial oven temperature was 80 °C, which was ramped to 300 °C with 30 °C/min ramp rate and then held for 9 min. The helium carrier gas was set to a flow rate of 0.684 mL/min. The transfer line temperature was set to 280 °C. The mass spectrometer scan range was *m*/*z* 40–500, with a 2 min solvent delay and a scan rate of 1472 Da/s. The EI source temperature was 230 °C and the quadrupole temperature was 150 °C. Most of the replicate spectra of cocaine and its diastereomers from the NIST archive come from a select number of laboratories dating back to the 1980s, including the NYC Police Laboratory (P. Shah), the NIST MS Data Center, the Defense and Civil Institute for Environmental Medicine, Canada (J. Zamecnik), the Georgia Bureau of Investigation (P. Price), and the Virginia Department of Forensic Science. Additional laboratory sources for cocaine spectra in the SWGDRUG database are provided on the SWGDRUG website.^124^ Specific instruments and operating conditions of the database spectra are not known to us.

## Results and Discussion

### Kinetic Basis for General Linear Modeling

When a particular organic molecule is ionized using electron- or photoionization, the observed branching ratios, that is, the abundance of peaks in the resulting mass spectrum, is determined by four main factors:^125−127^ (1) the internal energy distribution of the molecule prior to ionization, (2) the excitation energy distribution accompanying ionization, (3) the apparent reaction time or observation time that is specific to the apparatus, and (4) mass bias and spectral distortion caused by ion optics and instrument conditions.^88,128^ Branching ratios may be further affected by collisions between activated ions and residual gases en route to detection.

The relationship between the internal energy distribution of an activated ion and the branching ratios of its product ions can be explained by classical statistical models such as the quasi-equilibrium theory (QET)^127^ or the Rice–Ramsperger–Kassel–Marcus (RRKM) theory of unimolecular dissociation.^127,129−133^ These theories pose that the reaction rate of a particular pathway is determined by the entropy and enthalpy of activation of the transition state,^129,134,135^ and they are sufficiently reliable to enable EI fragmentation patterns to be accurately modeled and reasonably well predicted from first principles.^127,136^ Bauer and Grimme provide an excellent account of the rich history in this area.^136^

**Figure 1:**
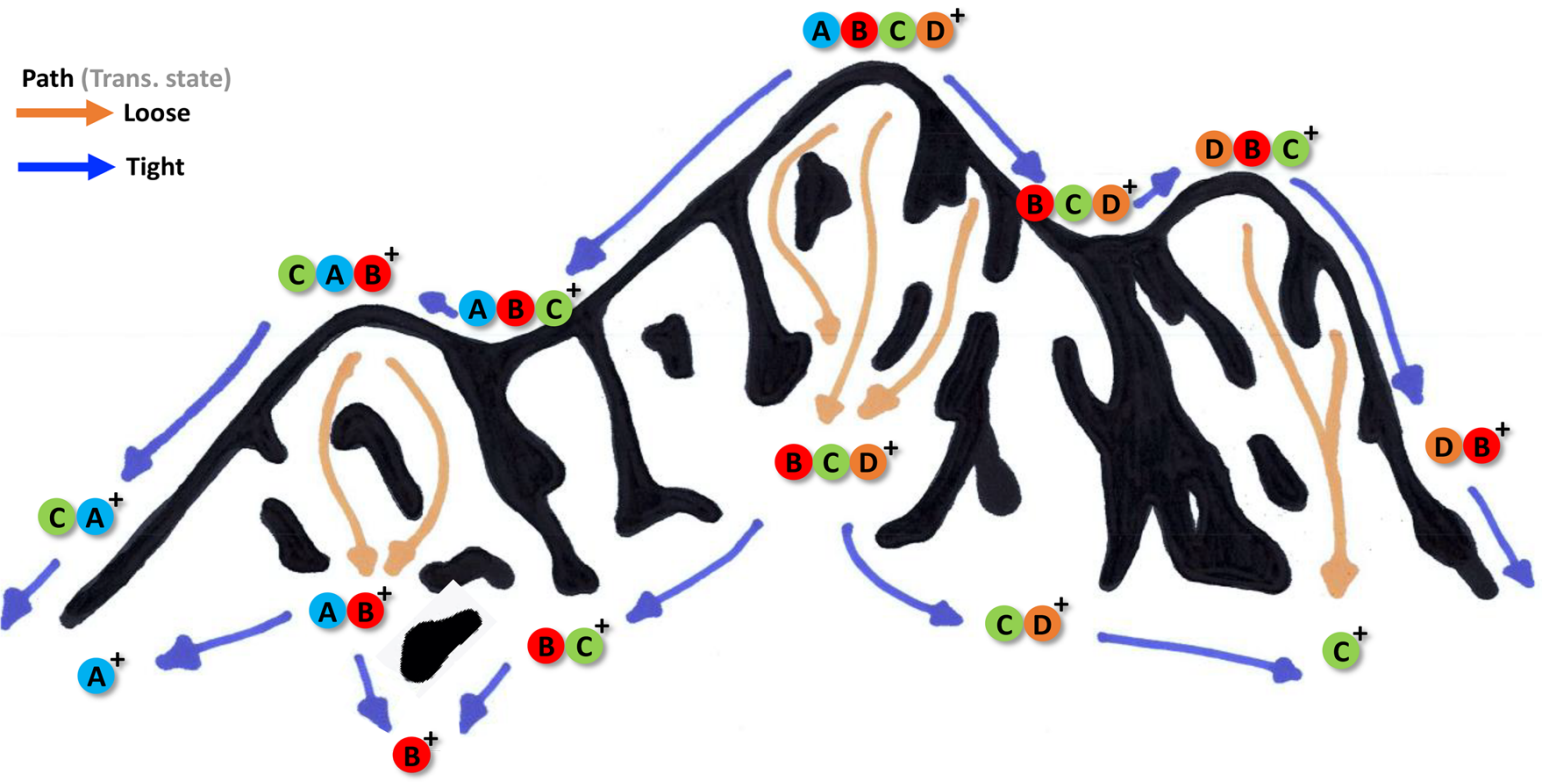
Schematic visualization of different potential energy pathways down an energy landscape. Tight transitions states can be thought of as narrow paths that take time to traverse and tend to involve rearrangements. Loose transition states can be thought of as wide-open pathways falling sharply off the mountain; they are faster and more numerous but can only be accessed from higher states.

One way to appreciate the mathematical relationship between the dissociation rate and the density of transition states is through the Kassel equation,^137,138^ which shares general trends with the more-sophisticated QET and RRKM theories but is easier to understand.^125,135^ The flow of different ions through multicoordinate transition space can be thought of as a precursor ion having many different pathways down a molecular landscape, as shown in Figure 1. In this analogy, certain elevations, and therefore certain pathways, are not accessible if the excitation energy is too low that the precursor can only ascend part-way up the mountain.

The Kassel equation (eq 1) assumes that the rate of a reaction as a function of energy *k*(*E*) is the product of the frequency factor of a certain energy level and the probability of populating the energy level:
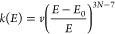
1where *v* is an entropic factor or frequency factor, proportional to , that describes the tightness of the transition state. The term  describes the fractional probability of populating an energy level *E* above the critical energy *E*_0_. As the internal energy of the precursor ion increases, the fraction  approaches unity. The term *N* is the number of atoms in the precursor, so the power term 3*N* – 7 is the number of vibrational degrees of freedom in a nonlinear precursor (not including the reaction co-ordinate).

A schematic visualization of Kassel’s equation is shown in a potential energy hypersurface in Supplemental Figure S2. Kassel’s equation explains how the dissociation rate of a reaction increases when the entropy is more favorable, when the activation energy increases, or when the number of atoms is smaller. The corollary is that larger ions require larger internal energies to observe even the lowest energy rearrangements, a phenomenon which has hindered the effectiveness of CID of high-mass ions in top-down proteomics.^139−141^

When millions of activated precursor ions are formed in an EI source at any given instant, they start with a distribution of internal energies, or elevations up the mountain. Ions can either follow loose transitions, represented by steep, fast, and unspecific pathways down the mountain, or tight transitions, represented by less steep, slower, and specific pathways down the mountain. The ions end their paths with frequencies of occurrence in proportion to their statistical probabilities. At short observation times, the only fragments that can be observed are those that transition through fast or loose transition states. At longer observation times, fragments occurring through longer or tighter transition states become more prominent. Below, we illustrate how these basic principles of QET/RRKM transition state theory lead to approximately linear fragment-fragment correlations that can be effectively fitted with general linear models to extrapolate the kinetic-based behavior between instruments. To be clear, QET/RRKM theory does not result in a mathematically rigorous proof of linear branching ratios. Instead, basic modeling is used to show that when the activation energy vastly exceeds the appearance energies of the different pathways, then modest changes of ∼30% in the internal energy or 50% in the reaction time will provide some approximately linear relationships between at least several pairs of ions. In practice, the method of mixed stepwise selection of general linear modeling can effectively identify and employ the ions that best correlate with one another among the replicate spectra to build the general linear models (GLMs). Neither human selection nor knowledge about the structural relationship between different ions is required for successful implementation of EASI.

### Simulated Kinetics and Branching Patterns

**Figure 2:**
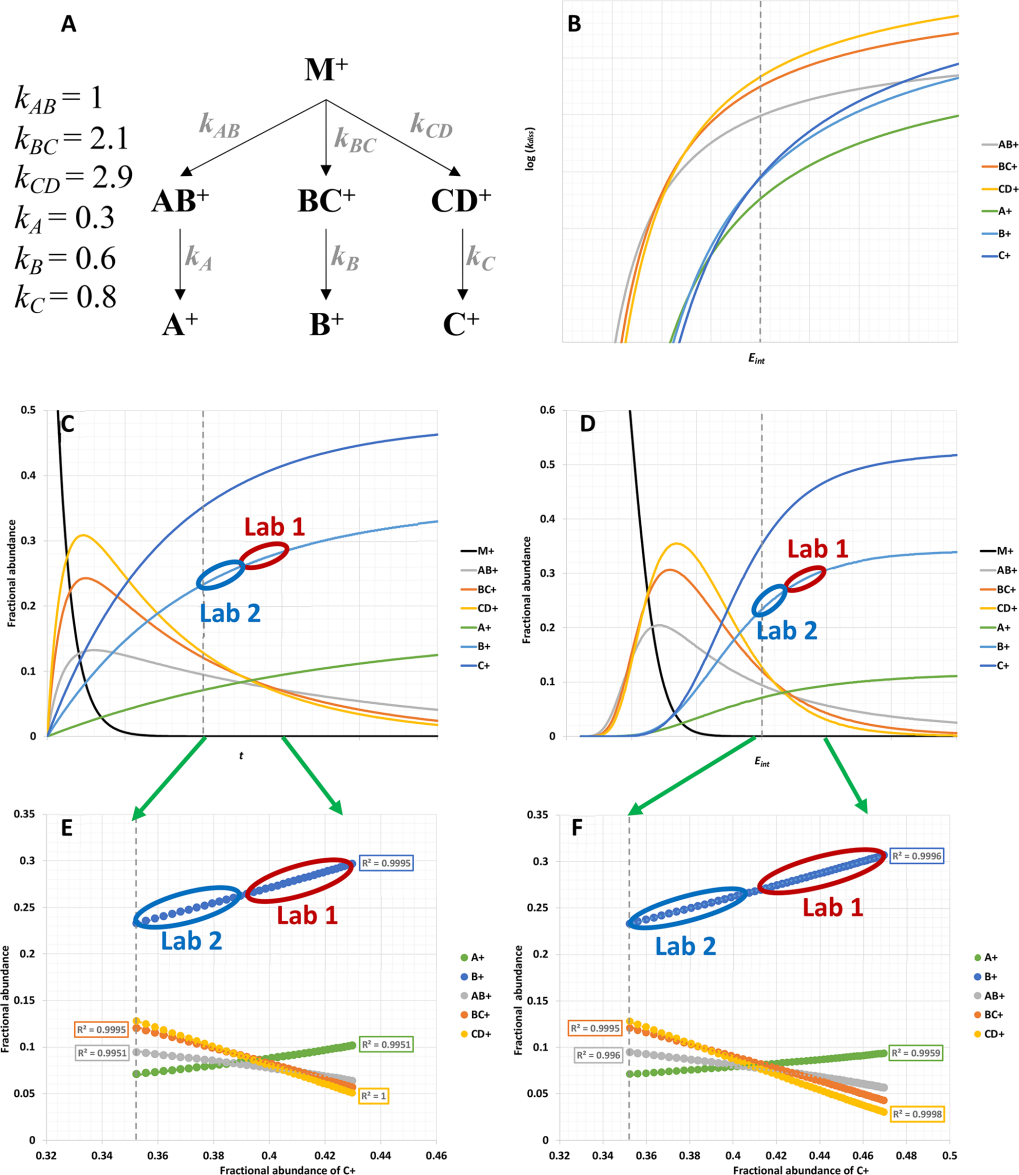
Theoretical modeling to show how changes in fractional ion abundances as a function of internal energy and observation time can be linearly extrapolated between instruments: (A) modeled system and relative rates of fragmentation at the vertical dashed line in each panel; (B) modeled log(*k*_*diss*_) versus internal energy at a fixed reaction time; (C) breakdown curves as a function of time at a fixed internal energy; (D) breakdown curves as a function of internal energy at fixed time; (E) ion abundances versus the abundance of C^+^ over the range of times in panel C; and (F) ion abundances versus the abundance of C^+^ over the range of internal energies in panel D. One grid width in Figures B, C, and D spans a 33% increase in internal energy and 50% increase in reaction time relative to the dashed line for the conditions in panel A.

Consider a precursor molecular ion M^+^ with the generic structure ABCD^+^. QET/RRKM theory can apply to both odd- and even-electron ions, so a radical is not shown for simplicity. Figure 2 shows a hypothetical branching pattern of a simple molecule that includes both competitive and sequential fragmentations. For simplicity, the modeled rates have been normalized to the rate of *k*_*AB*_ at a given arbitrary internal energy (*E*_*int*_) and observation time (*t*), as provided in Figure 2A. The modeling is based on a hypothetical precursor with 70 atoms, arbitrary activation energies between 1.6 and 3 eV and arbitrary frequency factors. The modeling further assumes that the internal energy of a precursor is distributed between the free energy of the pathway and the internal energy of the fragment ions. The fractional ion abundances (assuming [*M*^+^]_*t*=0_ = 1) as a function of time (*t*) were derived from a combination of branching rates and consecutive rates according to the following examples:^125,134−136^
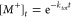
2where *k*_*tot*_ = *k*_*AB*_*+ k*_*BC*_*+ k*_*CD*_.
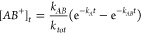
3
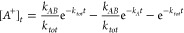
4

Figure 2A shows the modeled fragmentation pathway and Figure 2B shows the rate of change of log(*k*_*diss*_) versus internal energy *E*_*int*_ for the different pathways. The fragments in the key are listed in ascending order of activation energy and descending order of tightness of the transition state. Therefore, AB^+^ is the lowest energy rearrangement product and is observed at the lowest appearance energies.^134,135^

In Figure 2C–F, the fragment C^+^ has the highest appearance potential but the loosest transition state, so its rate increases more quickly with internal energy than the other pathways. The regions labeled “Lab 1” indicate a hypothetical range of measurements observed by one laboratory based on the variance in their data, which is characterized by the specific geometry of the lab’s instrument, the type of mass spectrometer, and their specific operating conditions. If Lab 2 uses a different geometry of instrument, such as from a different vendor, or uses different operating conditions, then the relative ion abundances observed in Lab 2 will differ from Lab 1 because of the different internal energies and observational timeframes of fragmentation. For example, in Figure 2C, the mean fractional abundance in Lab 1 for the fragment B^+^ is 0.270 ± 0.015% (95% confidence interval) and in Lab 2, the mean fractional abundance is 0.245 ± 0.015% (95% confidence interval). A Student’s *t*-test could easily show that the abundance of B^+^ is significantly different at *p* < 0.05, and any “good” algorithm that requires spectral similarity as a modus operandi should find most replicates from Lab 2 are significantly different from most replicates of Lab 1. Other fragment ions may also be significantly different between the two laboratories. These differences between instruments are limiting factors in the effectiveness of algorithms that use spectral similarity as a mechanism for compound identification.

In contrast to spectral similarity methods that are typically used to identify substances,^13,37,42,71^ the linear regression lines in Figure 2E,F indicate that the linear regression equations for Lab 1’s data can be extrapolated to predict ion abundances in regions of experimental space that are beyond the natural variance captured in Lab 1’s data. In other words, Lab 1 could extrapolate the trend in its data to predict ion abundances for data collected in Lab 2. Such capabilities are generally not possible when the variance within a training set does not capture the variance of the test or validation set, as demonstrated by the PCA plot in Supplemental Figure S3.

For an analyst in Lab 1 to make accurate predictions about fragment ion abundances in a spectrum of M^+^ in Lab 2, Lab 1 would only need to know the values of the covariate ion abundances (i.e., a measured spectrum from Lab 2) and the coefficients derived from the various regression models. Lab 1 would not need to know the source of the variance nor conduct any corrections or adjustments to Lab 2’s spectra to minimize the residual errors between the predicted and measured ion abundances. One caveat is that Lab 1’s spectral variance may be dominated by changes in excitation energy whereas a Lab 2’s spectra might differ from Lab 1 primarily because of a difference in apparent observation times. In such cases, if the rate of change in ion abundances as a function of time and energy are disparate, then the linear models based on changes in energy may not make accurate predictions about ion abundances affected by changes in time. Such issues could be answered and solved by using a training set that incorporates data from all the instruments of intended deployment.

Figure 2E,F indicates that, whether the variance between Labs 1 and 2 are caused by differences in internal energies or observation times, there are likely to be some strong linear relationships that relate the two domains of variance. EASI outlines a basic framework to help identify the ions that best explain one another’s abundance and thereby enable robust and accurate predictions between laboratories. Here, we use stepwise general linear modeling, but many other solutions to interlaboratory comparisons are conceivable based on the same underlying assumption of linear correlations between ion abundances of replicate spectra.

**Figure 3:**
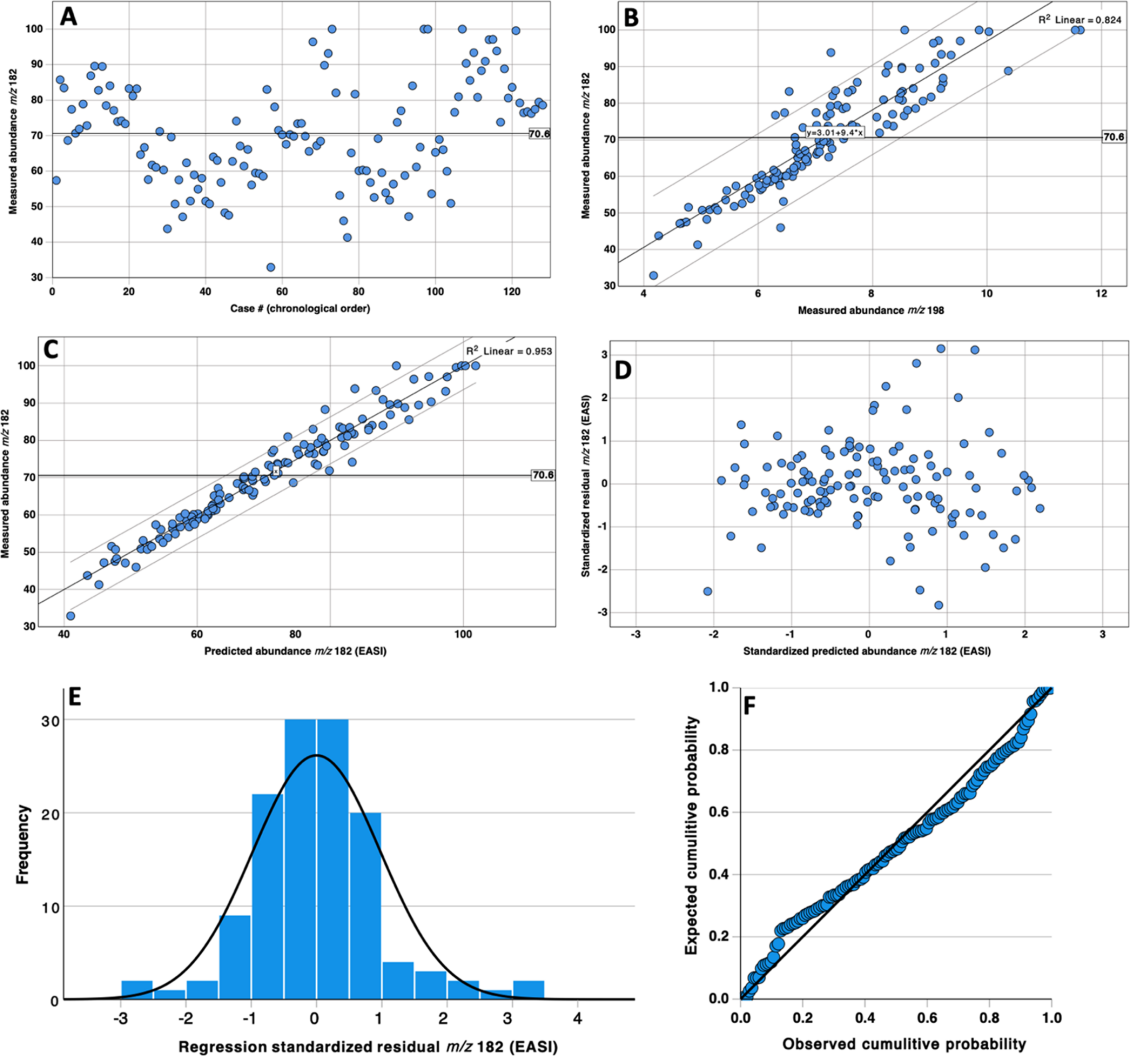
Scatter plots of measured and predicted ion abundances for *m*/*z* 182 for cocaine: (A) normalized abundance of *m*/*z* 182 relative to the base peak in 128 spectra (cases) collected over 6 months; (B) scatter plot of the normalized abundance of *m*/*z* 182 versus the normalized abundance of *m*/*z* 198 in the same data; (C) scatter plot of the normalized abundance of *m*/*z* 182 versus the EASI-predicted abundances using the coefficients shown in Table 2; (D) scatter plot of the standardized residuals versus the standardized predicted abundances based on the 128 predictions in panel C; (E) frequency distribution plot of the standardized residuals of the 128 predictions in panels C and D; and (F) P–P plot of the standardized residuals in panels C and D.

To gain an appreciation of the natural variance of correlations among measured product ions in replicate spectra, Figure 3A shows the relative abundance of *m*/*z* 182 versus case number for 128 replicate analyses of a cocaine standard in an operational forensic laboratory over a 6-month period.^28^ The database includes an average of about one cocaine spectrum per business day. Our previous analysis of the same data included a brief observation of the correlations between ion abundances in these replicate spectra, but the previous study did not address the source of the variance nor how to take advantage of the correlations to enable spectral identifications.^28^ When the normalized abundance of the peak at *m*/*z* 182 of cocaine is plotted as a function of the case number, the abundances vary over the wide range of ∼34–100% (Figure 3A). In cases where *m*/*z* 182 was the base peak, the abundance of *m*/*z* 82 was as low as 78%. Again, most past and present algorithms assume this scatter is random^48,142^ and/or that this variance can only be controlled using “properly tuned” instruments.^38,55^ However, the bivariate plot in Figure 3B shows that the variance in the normalized abundance of *m*/*z* 182 is not random and that, for example, ∼ 82% of the variance in its normalized abundance can be explained by the normalized abundance at *m*/*z* 198. This is to say that the abundance at *m*/*z* 182 correlates far better with the abundance at *m*/*z* 198 than it does with the peak at *m*/*z* 82, which is typically the base peak.

Table 1 shows the bivariate correlations for the 20 most abundant fragments in the training set of 128 cocaine spectra collected over 6 months in Lab 1. Admittedly, the choice to model the 20 most abundant ions is somewhat arbitrary. The number of variables could be optimized and validated in future work and with different substances. In this case, the choice to include 20 ion abundances deliberately enabled the inclusion of the fragments at *m*/*z* 94 and *m*/*z* 152, which are known to be important for the discrimination of cocaine from its diastereomers, like pseudococaine.^117,118^ The introduction includes many examples of algorithms that report only a slight loss in performance when using smaller fractions of the measured peaks in spectral comparison algorithms.^11^

**Table 1:**
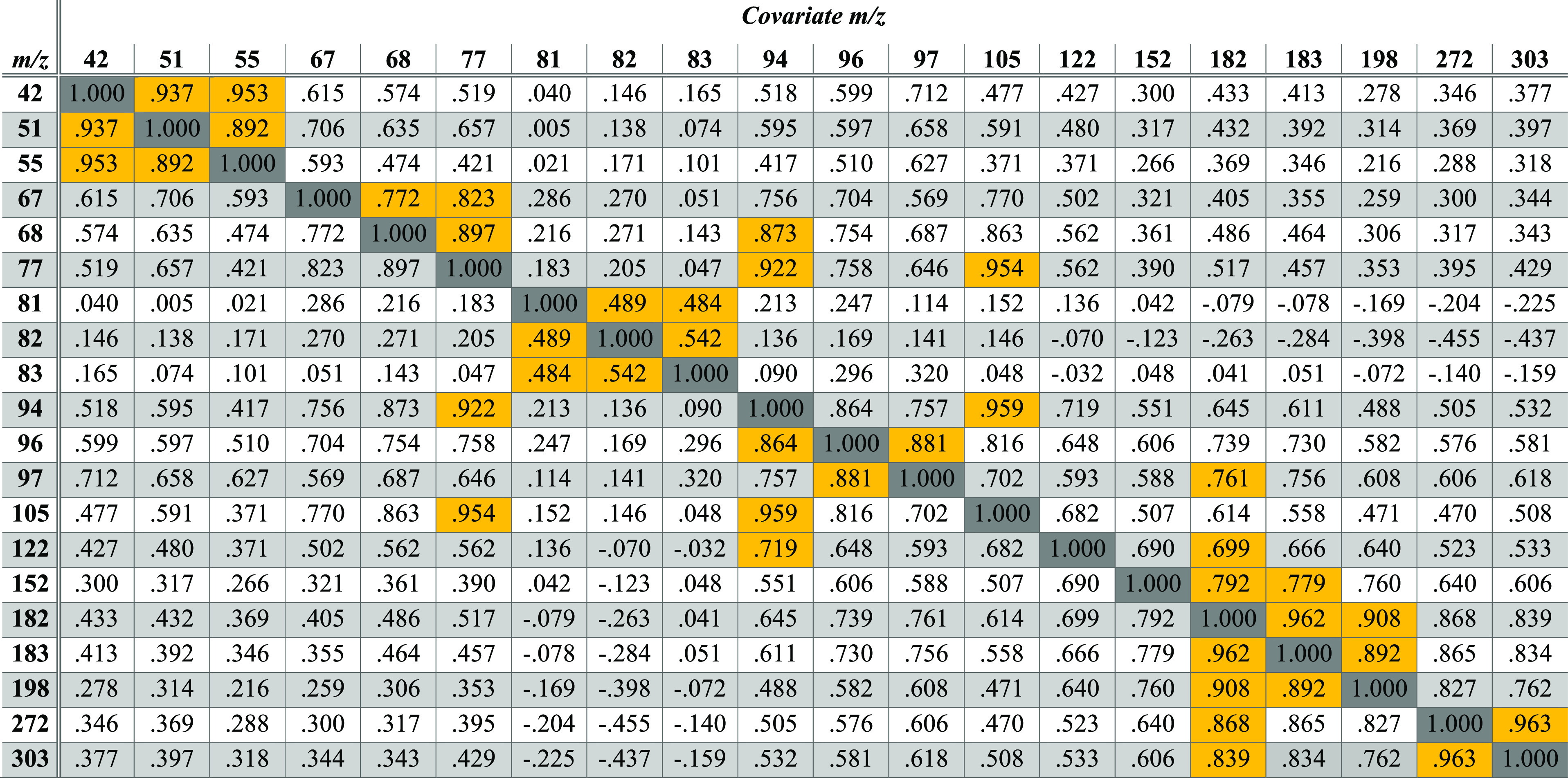
Summary of Bivariate Pearson Correlations for the 20 Most Abundant Fragments of Cocaine^a^

a Abundances were normalized to the base peak before analysis. *N* = 128 spectra over 6 months from an operational crime laboratory (Lab 1). The two largest bivariate Pearson correlations in each row are shaded gold.

The statistical significance of each cross-correlation is provided in Table S1. Table 1 shows that, in general, ions that appear close in *m*/*z* value to one another tend to correlate more strongly than with ions of disparate *m*/*z* values. This observation can be thought of as a mass bias or tuning effect, which causes ions of similar *m*/*z* to correlate. Exceptions are *m*/*z* 105 and *m*/*z* 77, which share a strong correlation by virtue of the close mechanistic and kinetic relationships between them, i.e., *m*/*z* 77 is a direct cleavage product of −CO (28 Da) from *m*/*z* 105.^116−118^ As shown in Table 1, the normalized abundance of *m*/*z* 198 visualized in Figure 3B is one of many ions that correlate strongly with the normalized abundance at *m*/*z* 182, so several other ions could also serve as independent variables with which to model or predict the abundance at *m*/*z* 182 as the dependent variable.

Extending this idea further, the abundance of *m*/*z* 182 as a dependent variable can be modeled or predicted using a general linear model by employing multiple covariates in a single model, as shown in eq 5:
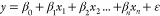
5where *y* is the abundance of the dependent variable (e.g., *m*/*z* 182), β_0_ is the *y*-intercept, β_1_ through β_*n*_ are the covariate coefficients, *x*_1_ through *x*_*n*_ are the covariate ion abundances (e.g., *m*/*z* 198 and *m*/*z* 183), and ε is the residual error. Conceptually, general linear modeling (GLM) can be thought of as a weighted average of more than one linear regression model (*y* = β_0_ + β_1_*x*_1_ + ε). GLM is a classical statistical technique that was elegantly demonstrated by Sir Francis Galton in 1886 when he showed that the height of an adult, the dependent variable, could be accurately modeled or predicted using the heights of his/her biological parents, the covariates.^143^ In an application vaguely similar to the one described here, GLM has also been used to estimate missing values (not *m*/*z* abundances) from a failed sensor to help stabilize the behavior of different multivariate models in a process-control setting.^144^ General linear modeling has also been used to predict GC retention indices as the dependent variable from a variety of molecular properties as covariates.^145^

In Figure 3C, the abundance of *m*/*z* 182 as the dependent variable was modeled in IBM’s SPSS software (version 28.0) using stepwise development of a general linear model. Covariates were added when their contribution to the model was significant at *F* ≤ 0.05 and removed when their contribution was insignificant at *F* ≥ 0.1. Of the 20 developed models, the models varied from as few as three covariates (not including the y-intercept) for *m*/*z* 272 to as many as 12 covariates to effectively model *m/*z 51. The unstandardized coefficients in the 20 GLM models are provided in Table 2. The fact that the stepwise models never require all 20 ions to maximize the extent of explainable variance is an indication that satisfactory performance of GLM could probably be obtained using fewer than 20 ion abundances at the onset. Again, the focus of the current manuscript is a proof-of-concept rather than complete optimization.

**Table 2:**
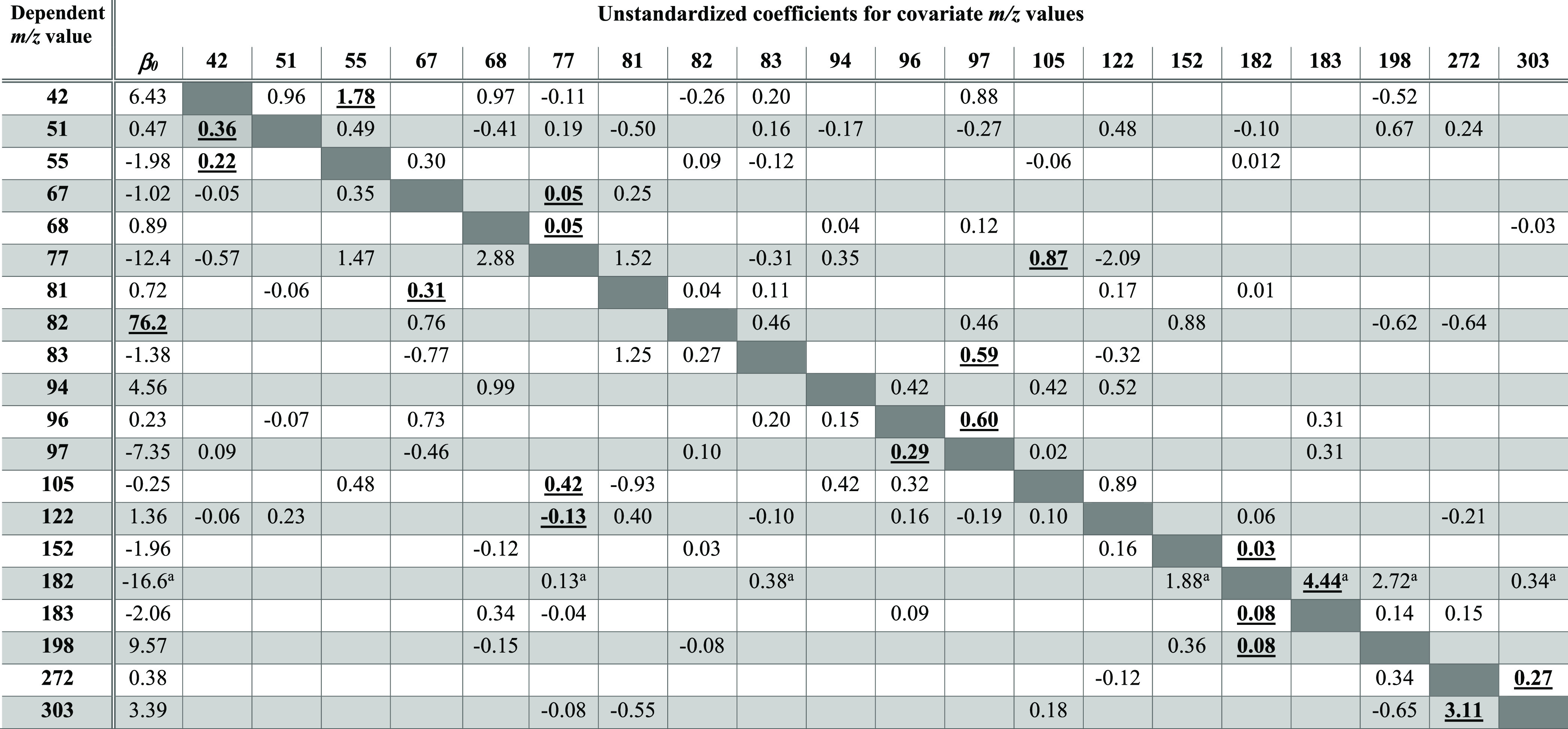
Summary of the Unstandardized Coefficients for 20 General Linear Regression Models Using the Abundance of Each *m/z* Value as a Dependent Variable and the Remaining 19 Abundances as Possible Covariates*

* Bold underlined font indicates the most significant standardized coefficient in each regression model. *N* = 128 spectra were used as the training set with the 20 most abundant peaks selected.

a As an example of how to use these coefficients, these coefficients are employed in eq 6 to predict the abundance of *m*/*z* 182 in any given query spectrum.

As an example of how to use this table, the abundance of *m*/*z* 182 (*Â*_182_) in any cocaine spectrum can be predicted using the remaining ion abundances in the spectrum using the equation:
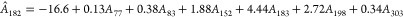
6

The results of 128 such predictions using this model are shown against the measured values for the training set in Figure 3C. The coefficient of determination (*R*^2^) of the regression line shows that this general linear model explains more than 95% of the variance in the measured abundances of *m*/*z* 182. Therefore, more than 95% of the variance in the abundance of *m*/*z* 182 in the 128 spectra is not random and is explainable through covariance mapping. Figure 3D–F shows different ways of assessing the distribution of the residual differences between the modeled (predicted) abundances and the measured abundances at *m*/*z* 182 for the training set. In short, the residuals follow a normal distribution, are not significantly biased or skewed, and do not display problematic kurtosis. The residuals were also assessed for the remaining 19 models, and only 4 of the 20 models contained statistically significant skew. Seventeen of the 20 models contained kurtosis greater than the margin of error (0.425), and most had kurtosis measures >3, which indicates that the residuals generally displayed leptokurtosis with wider tails than Gaussian. Therefore, several of the observed P–P plots showed some deviation from perfect normality, but the deviations were mild and considered satisfactory as a proof-of-concept of this approach.

Future work should continue to address the validity of the assumptions necessary to support the proposed statistical modeling. For example, given that normalized ion abundances can never be negative, the measured distributions might act more Poisson than Gaussian, so modeling methods could be adjusted accordingly. As a final measure of validity, we also performed a correlation analysis on the residuals (ε) that resulted from GLM. Covariance mapping of the residuals of the training set predictions showed that only ∼10% of the 190 pairwise comparison tests between the different *m*/*z* channels showed any significant correlation. Therefore, GLM effectively explained and removed almost all the cross-correlations that existed in the normalized abundances of the replicate spectra in the training set.

So far, the discussion has shown that the branching ratios of the 128 cocaine spectra in the training set contain ion abundances that are strongly linearly correlated, as approximated over modest changes in energy and time by QET/RRKM theories of unimolecular fragmentation. Also, that GLM is a reasonably valid approach that can explain more than 90% of the variance in normalized ion abundances. The next step is to demonstrate that linear models built on the training set from one instrument can be extrapolated to spectra collected on different instruments.

**Figure 4:**
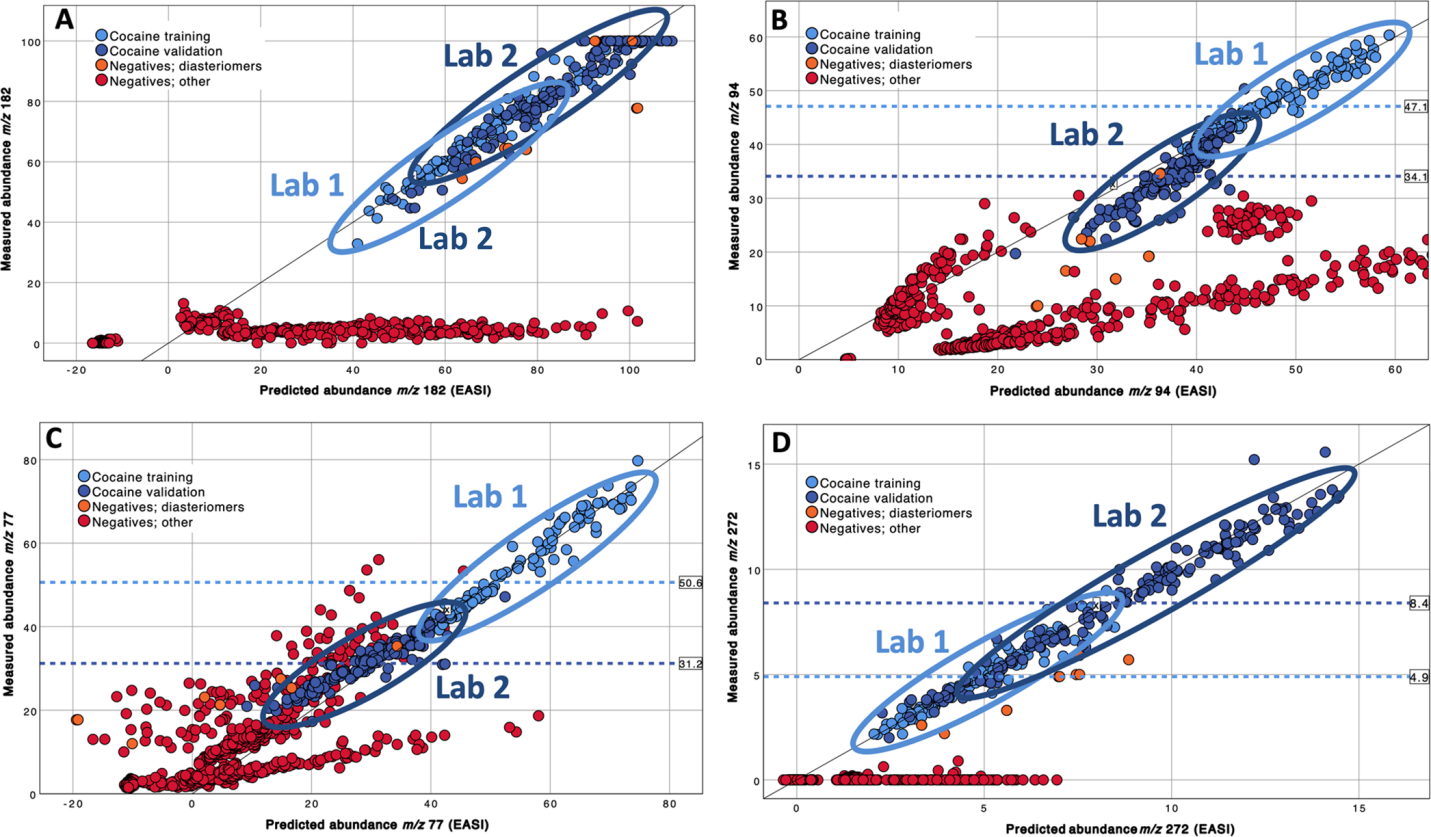
Scatter plots of measured and modeled/predicted values for a few selected ions of cocaine: (A) *m*/*z* 182, (B) *m*/*z* 94, (C) *m*/*z* 77, and (D) *m*/*z* 272. Horizontal lines show the mean abundances for the training set from Lab 1 (light blue) and validation set from other laboratories (dark blue). The line *y* = *x* refers to the ideal case of no residual error in predictions.

To demonstrate such extrapolation, the general linear models built on the training set of 128 cocaine spectra were applied to several groups of spectra: (1) a validation set that consisted of 120 validation cocaine spectra from a second crime laboratory (Lab 2); (2) 55 validation cocaine spectra from the NIST archive; (3) 706 replicate known negative spectra of ecgonine methyl ester, fentanyl, heroin, hydromorphone, and methamphetamine; and (4) a total of 10 replicate known negative spectra of the four diastereomers of cocaine from the NIST archive, including allococaine, pseudococaine, and pseudoallococaine. Bivariate plots of measured abundances versus predicted abundances for a select number of fragments of cocaine are provided in Figure 4 and Supplemental Figures S4–S8. Summary statistics for the four groups of spectra are also provided in Table S2.

Figure 4A is a scatterplot of the measured abundance of *m*/*z* 182 versus the predicted abundance of *m*/*z* 182 for all 1019 spectra in the database. The general linear model is the one shown in eq 6, but in this case the model is applied to all spectra in the database, including all known positives (KPs) and known negatives (KNs). The box and whisker plots in Supplemental Figure S9A show the same measured values in Figure 4A in a manner that makes the distributions of measured values easier to compare. Note that the KN diastereomers in orange provide measured abundances of *m*/*z* 182 that are closer to the mean of the training set (i.e., the consensus spectrum^57,121^) than most of the 175 KP cocaine validation spectra. For example, the mean abundances for *m*/*z* 182 are 70.6% for the KP training set and 72.7% for the KN diastereomers, but 88.4% for the KP validation set.

Most of the other KNs provide abundances for *m*/*z* 182 that are <10% relative abundance, so they are easily dismissed as not behaving like cocaine. Therefore, whereas all the measured abundances of *m*/*z* 182 in the spectra of cocaine and its diastereomers are notably different from the known negatives in the database, the relative abundances of *m*/*z* 182 of cocaine diastereomers are not significantly different (*t*-test, two-tailed, α = 0.05) from that of the cocaine training set.

Figure 4B is a scatterplot of the measured abundance of *m*/*z* 94 versus the predicted abundance of *m*/*z* 94 for all 1019 spectra in the database. Again, the general linear model was built on the training spectra of 128 cocaine spectra from Lab 1 and used the coefficients provided in the row for *m*/*z* 94 in Table 2. Box and whisker plots of the same data are shown in Supplemental Figure S9B. In this case, the training set has a mean abundance of 47.1% for *m*/*z* 94, the validation spectra of cocaine have a mean abundance of 34.1%, and the diastereomers have a mean abundance of 18.4%. The outlier in the diastereomers is a spectrum of pseudoallococaine, which has been shown to share spectral similarity with cocaine because of the steric similarity of the activated complexes.^117,118^ Here, the measured ranges of the training and validation sets of cocaine are significantly different (one-way ANOVA, post hoc least significant difference (LSD): *p* < 0.001). However, the regression line in Figure 4B enables accurate predictions for abundances of *m*/*z* 94 in the test set, even though the measured values in the test set are outside most of those in the training set. For example, the smallest abundance for *m*/*z* 94 in the training set of Lab 1 was 35.1%, but more than half of the test set provided abundances below this threshold. The ability to extrapolate the general linear model in Figure 4B to fit the abundance measurements of known positives made on different instruments and in different laboratories is unique to this algorithm, and it demonstrates the reliance on statistical behavior predicted by approximations of QET/RRKM theory, as demonstrated in Figure 2E,F.

The scatter plots of measured versus modeled abundances for *m/*z 77 and *m*/*z* 272 in Figure 4C,D further support the contention that the modeled linear behavior in one laboratory can be used to make accurate predictions about ion abundances in other laboratories, even when the range of measured values is substantially different between the laboratories. Supplemental Figures S4–S8 provide additional examples of effective extrapolations for fragments at *m*/*z* 68, 105, 122, 152, and 303, respectively. Each model varies in its effectiveness at extrapolating between the two laboratories. In many of these figures, the predicted abundances for the diastereomers of cocaine have larger residual errors than most of the cocaine spectra.

Although the current manuscript only provides modeling data for cocaine, we have applied GLM to replicate spectra of various analytes on different types of instruments, including: (1) electron ionization spectra from GC-MS instruments, (2) electrospray ionization tandem mass spectrometry (ESI-MS/MS) data from a quadrupole time-of-flight instrument, and (3) direct analysis in real time (DART) MS/MS spectra from a triple-quadrupole mass spectrometer. In all cases, multivariate linear models provided similar figures of merit as presented here, such as explaining more than 90% of the variance in the normalized fragment ion abundances, and that the residual errors between modeled and measured abundances were distributed in an approximately Normal manner. Furthermore, the residuals using EASI were typically ∼4 times smaller than residuals between measured abundances and their corresponding centroid values of the training set. Therefore, the results for cocaine presented here can be considered a typical result rather than an outlier.

These results indicate that instead of focusing on one or two specific ion ratios to enable discrimination between a compound and a closely related structure (e.g., cocaine and allococaine),^116−118^ GLM can help explain other sources of variance between cocaine and its diastereomers. There are many ways that one could use correlations between different pairs of ions in replicate spectra to establish whether the fragment ion abundances in a questioned spectrum follow the expected behavior of a substance or not. Several examples are described in part 2 of this manuscript.

## Conclusions

This manuscript demonstrates that GLM is a reasonably robust and valid approach to model the branching ratios of replicate spectra of cocaine. The residual differences between measured and modeled abundances for the 20 most abundant fragments of cocaine are approximately normally distributed and uncorrelated with one another, unlike the input normalized abundances. Most importantly, the models can be extrapolated to make accurate and robust predictions of relative ion abundances for cocaine spectra collected on various types of mass analyzers in different laboratories dating back to the 1980s. One example for the peak at *m*/*z* 182 shows that the variance in normalized ion abundance ranges from 40 to 100% (70 ± 30% confidence interval) in the training set, but a simple general linear model with one constant and six terms enables the same ion abundances to be predicted with confidence intervals less than ±2%. The linear behaviors modeled in this work are predicted by QET/RRKM theories of unimolecular fragmentation that were developed in the 1950s, and they are modeled using an approach that has been in use since at least the 1880s. In the future, neural networks and machine learning algorithms could also be used to take advantage of the linear relationships predicted by QET/RRKM theory. Finally, the developed coefficients for cocaine provided in Table 2 can be considered reasonably valid for predicting the relative ion abundances within any 70 eV mass spectrum of cocaine on any mass spectrometer, in perpetuity. To apply EASI to future casework samples of cocaine, analysts would not need access to a database of hundreds of replicate spectra of cocaine to enable reliable spectral identification, they would only need to refer to a table containing fewer than 100 linear coefficients, as provided in Table 2. The companion manuscript describes how to use the GLM models as a binary classifier to enable effective discrimination between cocaine and any known negatives, including its diastereomers.

## Supporting Information Available

3 tables and 9 figures, including: (1) normalized abundnaces for cocaine across a GC peak; (2) schematic visualization of a potential energy hypersurface; (3) summary statistics for 20 ion abundances of different groups of compounds; (4) PCA analysis results; (5) bivariate plots of GLM-predicted abundances versus measure abundances, and (6) box-and whisker plots of measured abundances for different data sets (PDF)
