# Supplementary material for: Expert Algorithm for Substance Identification Using Mass Spectrometry: Statistical Foundations in Unimolecular Reaction Rate Theory
Source: J Am Soc Mass Spectrom. 2023 May 31;34(7):1248–62. doi: 10.1021/jasms.3c00089 (PMC10326912; doi:10.1021/jasms.3c00089)
Supplement: Supplementary file 1 — js3c00089_si_001.pdf [file js3c00089_si_001.pdf]

Supporting Information

Expert Algorithm for Substance Identification (EASI) using Mass Spectrometry: Statistical Foundations in Unimolecular Reaction Rate Theory

Glen P. Jackson,[a,b*] *Samantha A. Mehnert,*[a,b,#] *J. Tyler Davidson,*[a,$] *Brandon D. Lowe,*[b] *Emily A. Ruiz,*[b] *Jacob R. King*[b]

[a]*Department of Forensic and Investigative Science, West Virginia University, Morgantown, WV, 26506, USA.*
[b]*C. Eugene Bennett Department of Chemistry, West Virginia University, Morgantown, WV, 26506, USA.*
[#]*Current Address: Department of Chemistry, Purdue University, West Lafayette, IN 47907, USA.*
[$]*Current Address: Department of Forensic Science, Sam Houston State University, Huntsville, TX, 77340, USA.*

[*]glen.jackson@mail.wvu.edu; *304-293-9236*

Sources of variance in experimental replicates

The practical limitations of even the most stable mass spectrometers prevent perfectly constant conditions, so replicate spectra always contain variance in their relative ion abundances. To show that uncontrolled changes in instrument conditions, can influence data on timescales of seconds, **Figure S3** shows a magnified region of a chromatogram of a cocaine standard collected between casework samples in an operational crime laboratory. When the fragment ion abundances are plotted as raw counts, all the ions of cocaine are strongly correlated (r>0.85), which is a necessary requirement for the successful application of most algorithms involving background-subtraction and peak-deconvolution.[1-10] However, after normalization to the base peak at *m/z* 82, the ions show a range of correlations with one another, from strongly positive (r=0.96) to strongly negative (r=-0.91). Again, the strong correlation with retention time indicates that the variance is closely correlated with a latent time-varying source of variance, such as a drift in pressure. The variance in the normalized abundance at each *m/z* value in each scan is not independently variable and not randomly variable, as is assumed/required by most comparison algorithms.

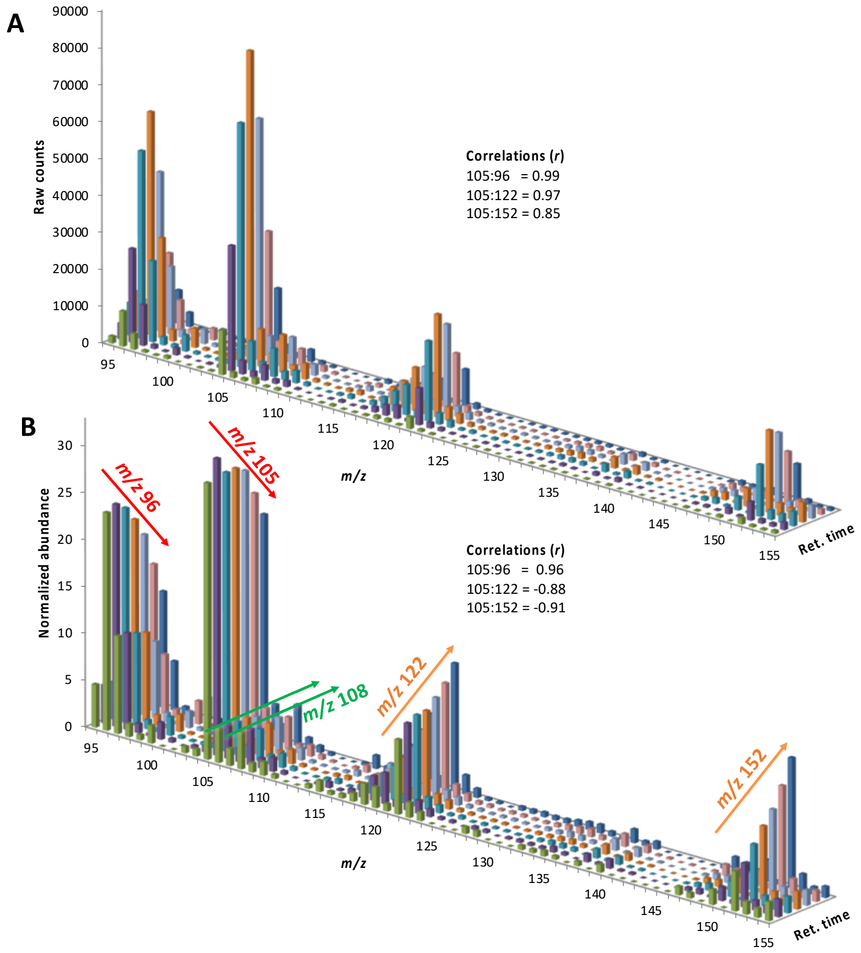

Figure S1. Magnified region of 3-dimensional GC-EI-MS data of cocaine to show: A) strong correlations between raw abundances (counts) of several fragments of cocaine as a function of time or scan number; and B) correlations (red-to-red and orange-to-orange), anticorrelations (red-to-orange) and weak/non-correlations (green-to-green) between normalized abundances of the same data in Panel A. Panel B demonstrates that normalized fragment ion abundances are not independently variable, as is often assumed.

Figure S2, adapted from Nishimura,[11] shows that fragmentation pathways involving molecular rearrangements require specific or 'tight' transition states that have lower activation barriers and are favored at the lowest internal energies. Such transitions can be thought of having low barriers and narrow pathways through co-ordinate space, like a narrow fast-flowing channel between two lakes.

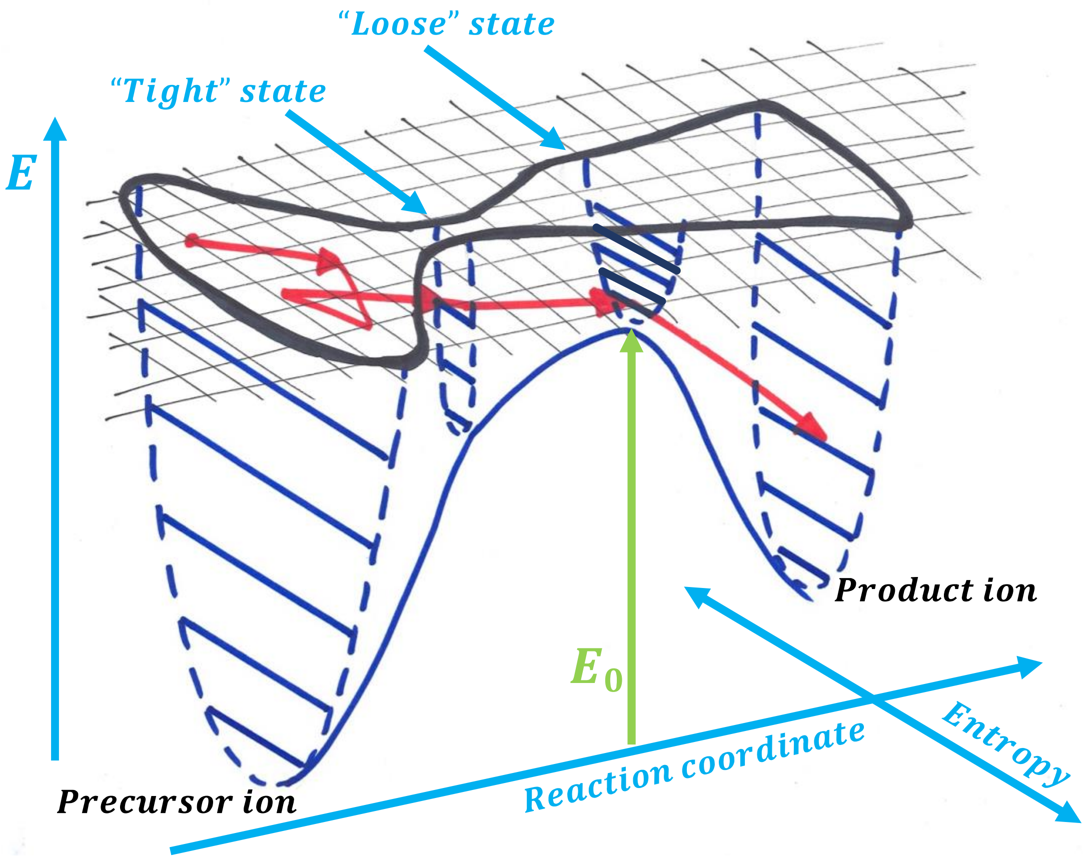

Figure S2: Schematic visualization of a potential energy hypersurface to highlight the effects of activation energy in the vertical dimension and activation entropy in the horizontal plane on the transition of an activated precursor ion through one reaction co-ordinate to a particular product ion. Fragmentation patterns derive from dozens of such reaction co-ordinates to dozens of product ions in a competitive manner.

In contrast, direct bond cleavages tend not to be sensitive to conformational requirements, have loose transition states, and tend to be favored at higher internal energies, like a wide slow-flowing river

between two lakes.[12,13] As the excitation energy rises, the looser transition states can ultimately carry a higher flux or volume of precursor ions to product ions. **Figure S2** helps visualize one reaction pathway, but dozens of reaction pathways are often possible for any given precursor ion. Each individual precursor ion will follow its own pathway through multi-coordinate space.

Table S1 shows the arbitrary values selected for the kinetic modeling example in Figure 2. The rates shown below are for an arbitrary time of 300 ns.

Property	M^+ (ABC^+)	AB^+	BC^+	CD^+	A^+	B^+	C^+
Relative rates		1.0	2.1	2.9	0.3	0.6	0.8
Diss. Rate (AU, Kassel)	53.3	17.0	36.3	49.4	5.7	11.0	13.6
Activation energy (E_0)		1.6	2.1	2.4	2.25	2.7	3
Frequency factor (v)		50	150	250	50	150	250
# Atoms	70	55	45	35	30	25	20
n (vib. DOF)	203	158	128	98	83	68	53

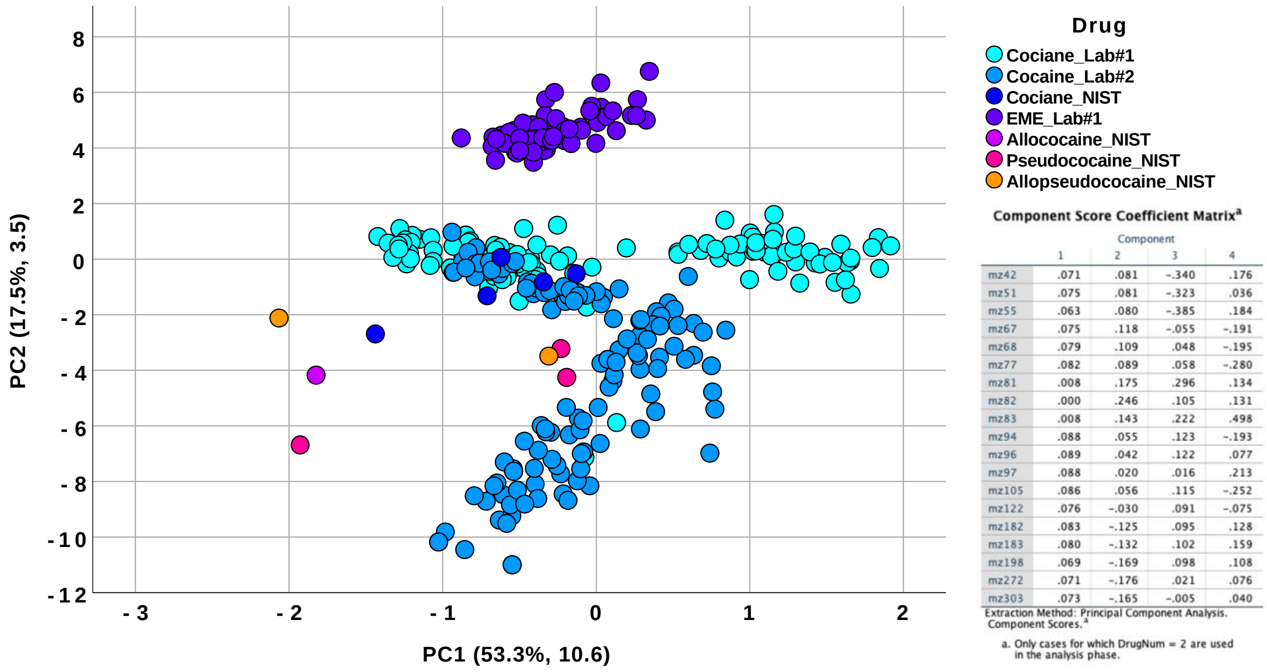

Component Score Coefficient Matrix[a]

	Component 1	2	3	4
mz42	.071	.081	-.340	.176
mz51	.075	.081	-.323	.036
mz55	.063	.080	-.385	.184
mz67	.075	.118	-.055	-.191
mz68	.079	.109	.048	-.195
mz77	.082	.089	.058	-.280
mz81	.008	.175	.296	.134
mz82	.000	.246	.105	.131
mz83	.008	.143	.222	.498
mz94	.088	.055	.123	-.193
mz96	.089	.042	.122	.077
mz97	.088	.020	.016	.213
mz105	.086	.056	.115	-.252
mz122	.076	-.030	.091	-.075
mz182	.083	-.125	.095	.128
mz183	.080	-.132	.102	.159
mz198	.069	-.169	.098	.108
mz272	.071	-.176	.021	.076
mz303	.073	-.165	-.005	.040

Extraction Method: Principal Component Analysis.
Component Scores.[a]

a. Only cases for which DrugNum = 2 are used in the analysis phase.

Figure S3. Plot of the first two principal components from PCA analysis of the fragment ion abundances of the 20 most abundant fragments in a database of 128 cocaine spectra from Lab 1 as the training set. PC2 correlates most strongly (and negatively) with the abundance of ions at *m/z* 182 and greater. Lab #1 usually has *m/z* 82 as the base peak and Lab #2 usually has *m/z* 182 as the base peak, which explains why the spectra from lab #2 fall so far outside the variance explained by the training set. Ecgonine methyl ester lacks many of the high mass ions of cocaine, which explains the positive displacement in the PC2 axis relative to cocaine.

Table S2. Significance of the coefficients in **Table 2** (1-tailed).

**Dependent *m/z* value**	**Unstandardized coefficients for covariate *m/z* values**																			
	**42**	**51**	**55**	**67**	**68**	**77**	**81**	**82**	**83**	**94**	**96**	**97**	**105**	**122**	**152**	**182**	**183**	**198**	**272**	**303**
**42**	.	<.001	<.001	<.001	<.001	<.001	.325	.050	.032	<.001	<.001	<.001	<.001	<.001	<.001	<.001	<.001	<.001	<.001	<.001
**51**	.000	.	.000	.000	.000	.000	.480	.061	.204	.000	.000	.000	.000	.000	.000	.000	.000	.000	.000	.000
**55**	.000	.000	.	.000	.000	.000	.408	.027	.129	.000	.000	.000	.000	.000	.001	.000	.000	.007	.000	.000
**67**	.000	.000	.000	.	.000	.000	.001	.001	.286	.000	.000	.000	.000	.000	.000	.000	.000	.002	.000	.000
**68**	.000	.000	.000	.000	.	.000	.007	.001	.054	.000	.000	.000	.000	.000	.000	.000	.000	.000	.000	.000
**77**	.000	.000	.000	.000	.000	.	.019	.010	.298	.000	.000	.000	.000	.000	.000	.000	.000	.000	.000	.000
**81**	.325	.480	.408	.001	.007	.019	.	.000	.000	.008	.002	.100	.044	.062	.319	.189	.191	.029	.010	.005
**82**	.050	.061	.027	.001	.001	.010	.000	.	.000	.062	.028	.056	.050	.215	.084	.001	.001	.000	.000	.000
**83**	.032	.204	.129	.286	.054	.298	.000	.000	.	.155	.000	.000	.296	.361	.295	.324	.284	.209	.057	.036
**94**	.000	.000	.000	.000	.000	.000	.008	.062	.155	.	.000	.000	.000	.000	.000	.000	.000	.000	.000	.000
**96**	.000	.000	.000	.000	.000	.000	.002	.028	.000	.000	.	.000	.000	.000	.000	.000	.000	.000	.000	.000
**97**	.000	.000	.000	.000	.000	.000	.100	.056	.000	.000	.000	.	.000	.000	.000	.000	.000	.000	.000	.000
**105**	.000	.000	.000	.000	.000	.000	.044	.050	.296	.000	.000	.000	.	.000	.000	.000	.000	.000	.000	.000
**122**	.000	.000	.000	.000	.000	.000	.062	.215	.361	.000	.000	.000	.000	.	.000	.000	.000	.000	.000	.000
**152**	.000	.000	.001	.000	.000	.000	.319	.084	.295	.000	.000	.000	.000	.000	.	.000	.000	.000	.000	.000
**182**	.000	.000	.000	.000	.000	.000	.189	.001	.324	.000	.000	.000	.000	.000	.000	.	.000	.000	.000	.000
**183**	.000	.000	.000	.000	.000	.000	.191	.001	.284	.000	.000	.000	.000	.000	.000	.000	.	.000	.000	.000
**198**	.001	.000	.007	.002	.000	.000	.029	.000	.209	.000	.000	.000	.000	.000	.000	.000	.000	.	.000	.000
**272**	.000	.000	.000	.000	.000	.000	.010	.000	.057	.000	.000	.000	.000	.000	.000	.000	.000	.000	.	.000
**303**	.000	.000	.000	.000	.000	.000	.005	.000	.036	.000	.000	.000	.000	.000	.000	.000	.000	.000	.000	.

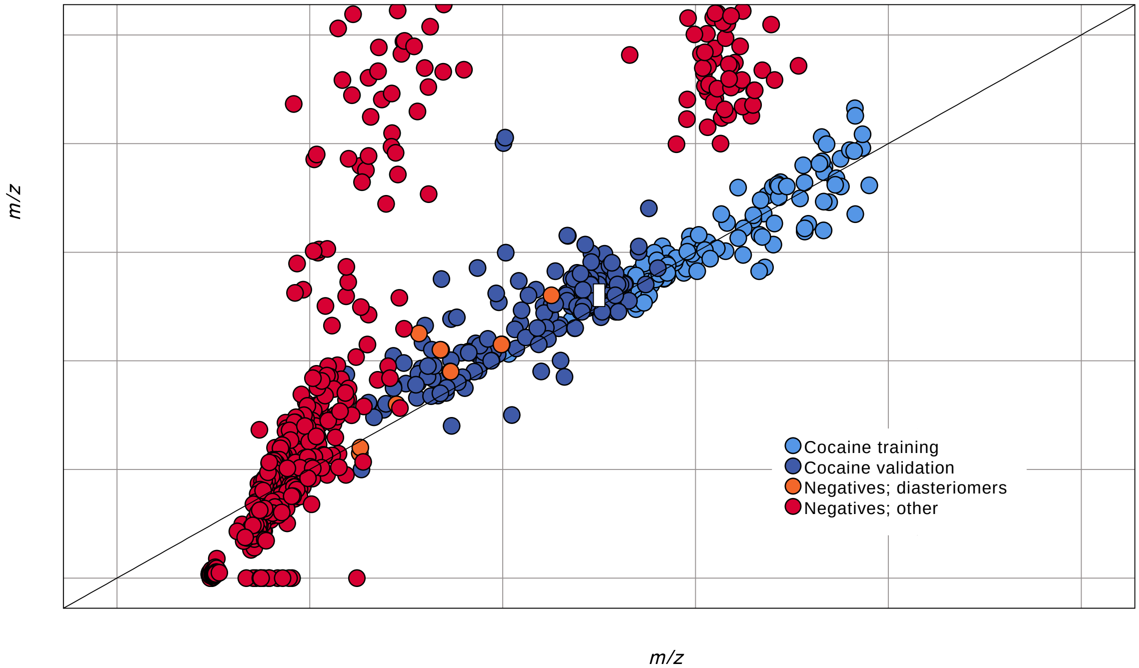

Figure S4. Bivariate plot of the measured abundance of *m/z* 68 versus the predicted abundance of *m/z* 68 for various drugs. The general linear model was built on a training set of 128 cocaine spectra from Lab 1 and applied to 175 validation spectra of cocaine, 10 diastereomer replicates (known negatives) and 706 additional known negative spectra.

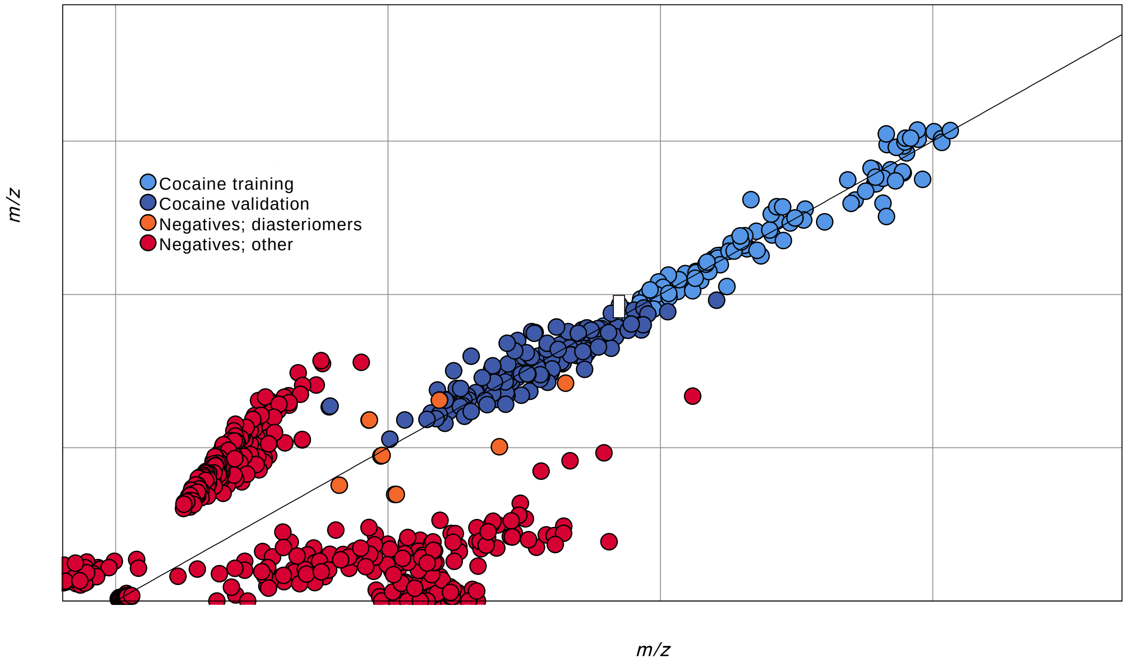

Figure S5. Bivariate plot of the measured abundance of *m/z* 105 versus the predicted abundance of *m/z* 105 for various drugs. The general linear model was built on a training set of 128 cocaine spectra from

Lab 1 and applied to 175 validation spectra of cocaine, 10 diastereomer replicates (known negatives) and 706 additional known negative spectra.

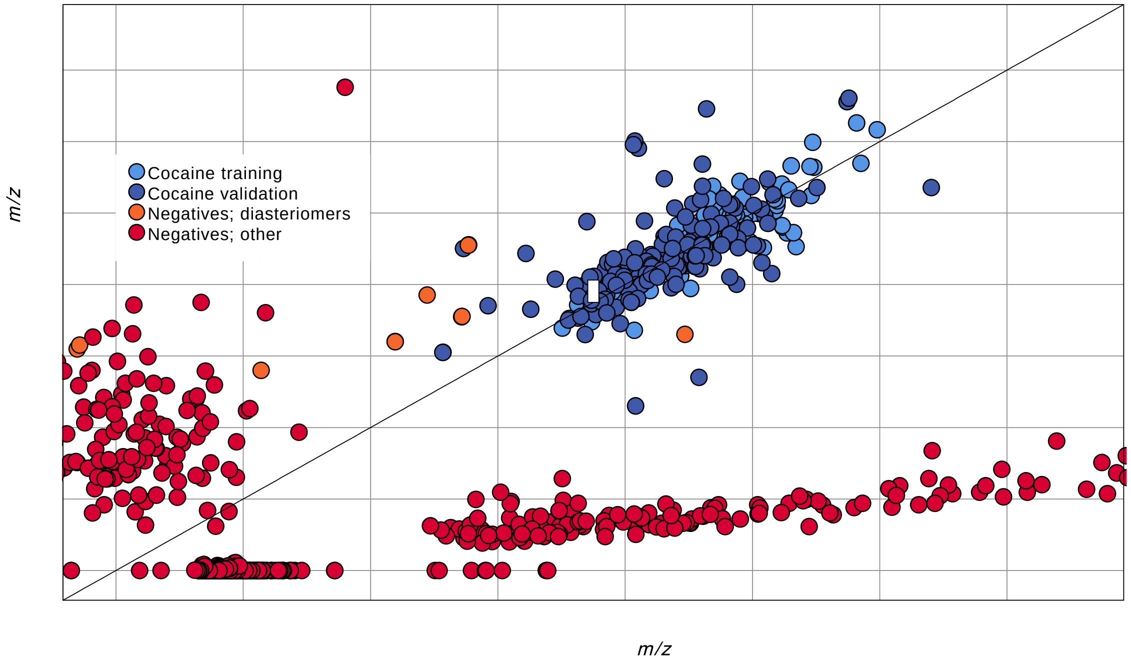

Figure S6. Bivariate plot of the measured abundance of *m/z* 122 versus the predicted abundance of *m/z* 122 for various drugs. The general linear model was built on a training set of 128 cocaine spectra from Lab 1 and applied to 175 validation spectra of cocaine, 10 diastereomer replicates (known negatives) and 706 additional known negative spectra.

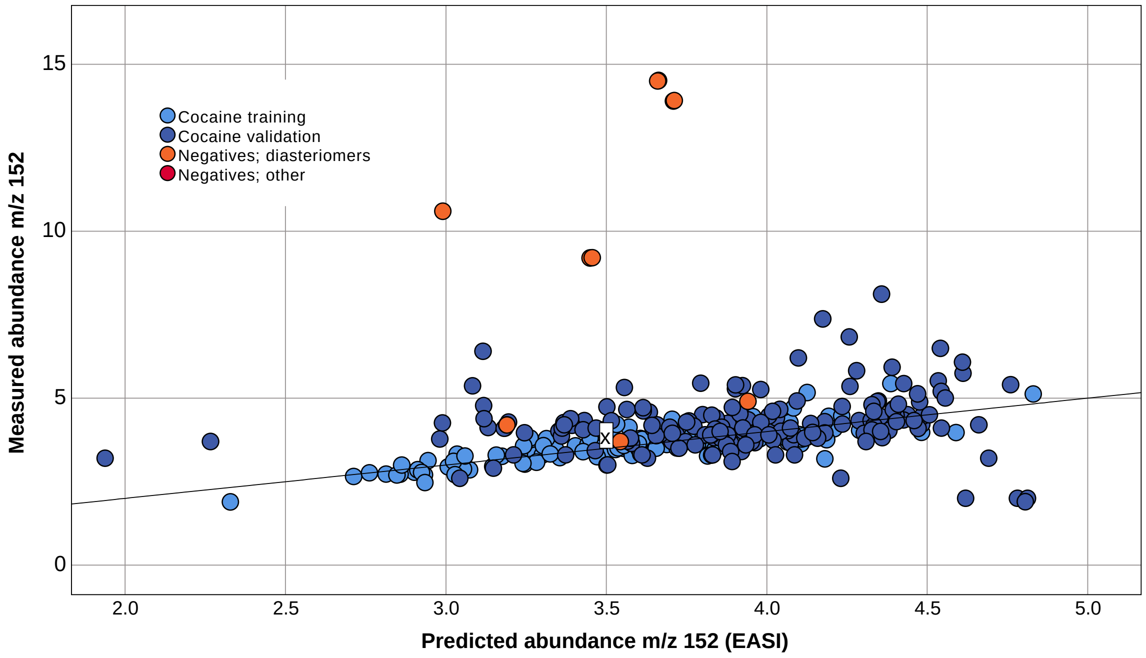

Figure S7. Bivariate plot of the measured abundance of *m/z* 152 versus the predicted abundance of *m/z* 152 for various drugs. The general linear model was built on a training set of 128 cocaine spectra from

Lab 1 and applied to 175 validation spectra of cocaine, 10 diastereomer replicates (known negatives) and 706 additional known negative spectra.

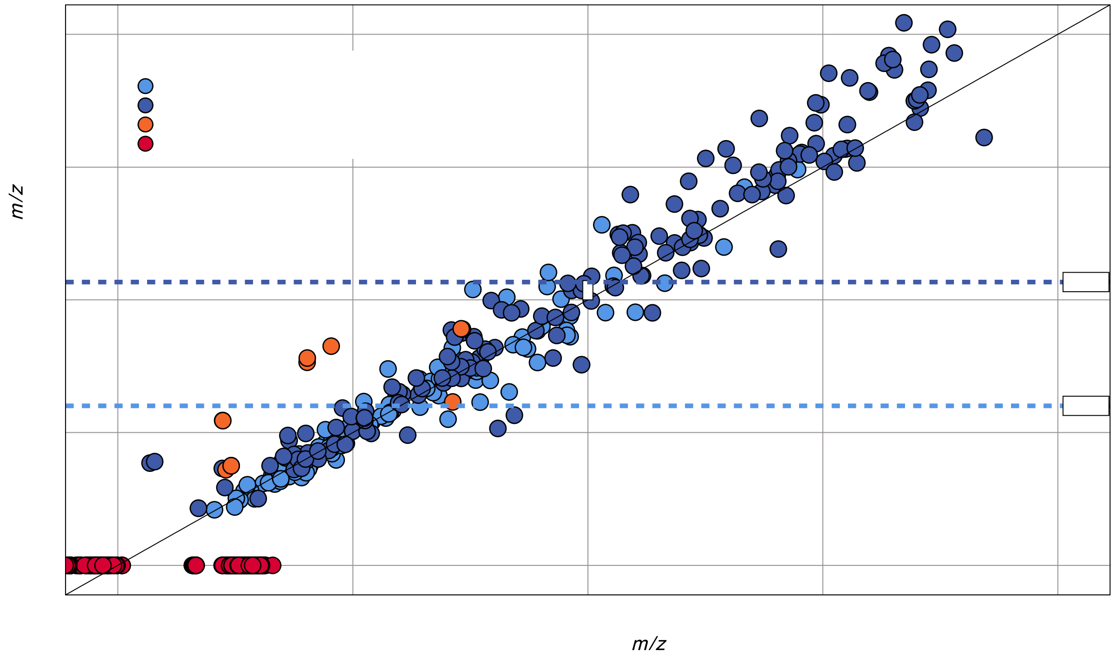

Figure S8. Bivariate plot of the measured abundance of *m/z* 303 versus the predicted abundance of *m/z* 303 for various drugs. The general linear model was built on a training set of 128 cocaine spectra from Lab 1 and applied to 175 validation spectra of cocaine, 10 diastereomer replicates (known negatives) and 706 additional known negative spectra.

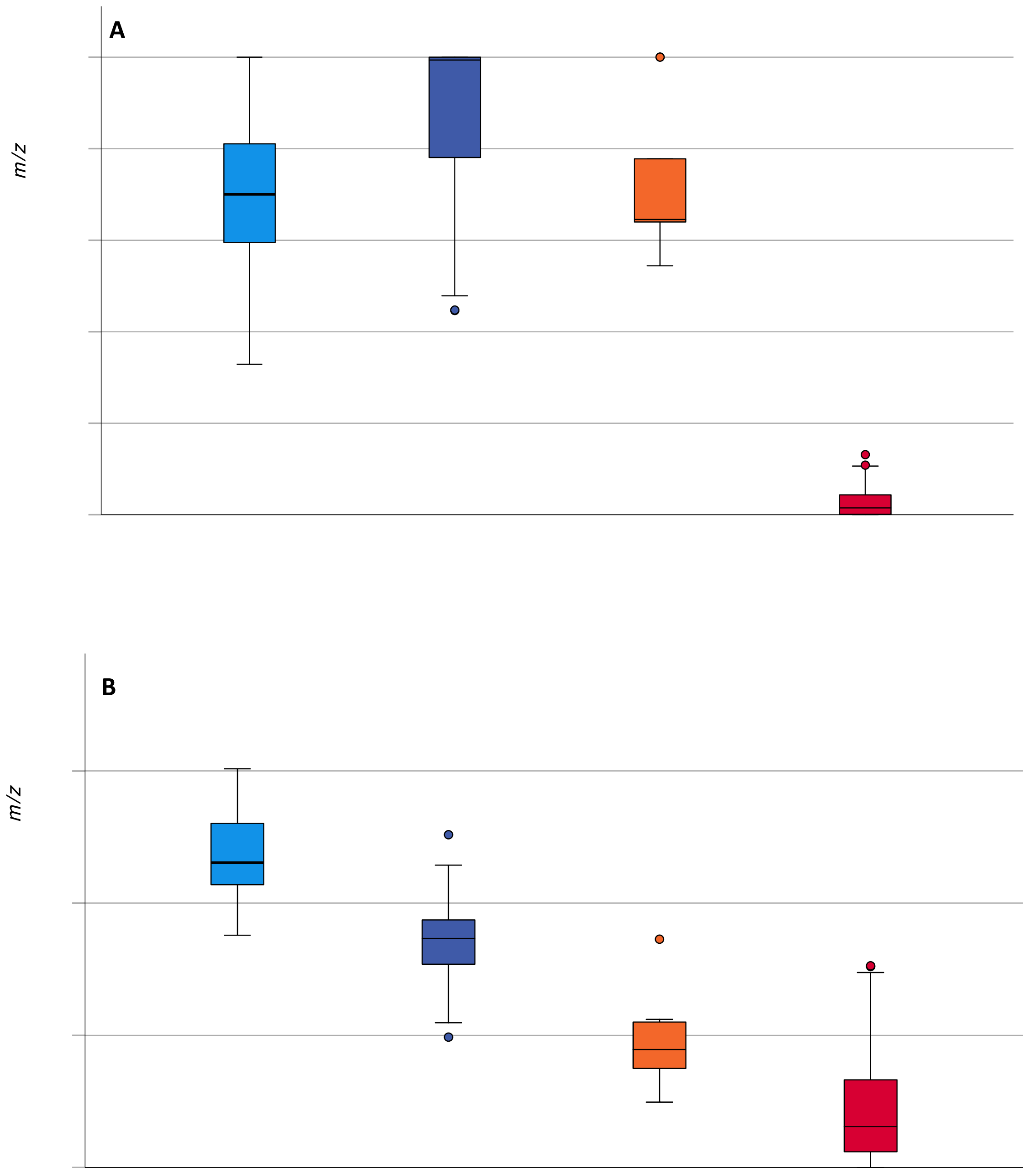

Figure S9. Box and whisker plots of the measured normalized abundance of *m/z* 182 (A) and *m/z* 94 (B) for different sets of drugs.

Table S3. Summary statistics for 20 ion abundances of different groups of compounds.

Report

Set#_4		mz42	mz51	mz55	mz67	mz68	mz77	mz81	mz82	mz83	mz94	mz96	mz97	mz105	mz122	mz152	mz182	mz183	mz198	mz272	mz303
Cocaine training	Mean	16.46	9.31	5.80	5.52	6.21	50.64	10.94	99.69	39.90	47.05	29.50	13.54	45.21	9.21	3.62	70.60	7.98	7.19	4.88	12.02
	N	128	128	128	128	128	128	128	128	128	128	128	128	128	128	128	128	128	128	128	128
	Std. Deviation	7.11	3.78	1.67	.86	.91	12.07	.53	2.42	1.77	5.91	2.70	1.67	8.46	1.10	.54	14.59	1.70	1.41	1.81	5.48
Cocaine validation	Mean	16.50	8.19	5.84	4.58	4.79	31.17	10.15	91.01	35.42	34.12	25.16	11.62	30.70	8.81	4.23	88.43	10.10	10.59	8.38	21.34
	N	175	175	175	175	175	175	175	175	175	175	175	175	175	175	175	175	175	175	175	175
	Std. Deviation	4.80	2.11	1.48	1.16	.91	5.04	1.91	12.65	5.63	5.19	3.40	1.87	3.74	1.25	.87	15.24	1.57	2.14	3.14	9.70
Negatives; diasteriomers	Mean	14.81	5.09	4.90	2.93	3.84	23.28	6.29	96.90	37.31	18.38	22.10	9.27	21.01	7.12	9.86	72.70	7.53	9.59	4.21	12.85
	N	10	10	10	10	10	10	10	10	10	10	10	10	10	10	10	10	10	10	10	10
	Std. Deviation	7.43	2.87	.85	1.78	.93	6.52	4.56	8.26	4.90	7.15	3.22	1.55	5.41	1.19	4.37	16.06	1.69	3.19	1.39	4.34
Negatives; other	Mean	24.45	3.67	7.21	2.92	4.49	11.28	6.40	17.57	8.26	8.69	22.50	5.25	8.11	1.48	4.12	2.48	1.33	3.31	.01	.00
	N	706	706	706	706	706	706	706	706	706	706	706	706	706	706	706	706	706	706	706	706
	Std. Deviation	22.23	3.35	8.24	2.62	5.33	10.05	7.97	29.79	16.34	8.18	25.59	10.03	7.68	1.99	5.35	2.66	1.59	4.98	.05	.00
Total	Mean	21.99	5.17	6.77	3.53	4.75	19.75	7.61	41.28	17.18	17.97	23.83	7.43	16.78	3.76	4.14	26.49	3.73	5.11	2.10	5.30
	N	1019	1019	1019	1019	1019	1019	1019	1019	1019	1019	1019	1019	1019	1019	1019	1019	1019	1019	1019	1019
	Std. Deviation	19.15	3.96	6.94	2.46	4.50	16.89	6.95	43.79	19.29	16.33	21.50	9.04	15.48	3.87	4.53	37.39	3.99	5.14	3.60	9.48

References:

(1) Dromey, R. G.; Stefik, M. J.; Rindfleisch, T. C.; Duffield, A. M., Extraction of Mass-Spectra Free of Background and Neighboring Component Contributions from Gas Chromatography Mass Spectrometry Data, *Anal. Chem.* **1976**, *48*, 1368-1375.

(2) Kim, S.; Koo, I.; Jeong, J.; Wu, S.; Shi, X.; Zhang, X., Compound identification using partial and semipartial correlations for gas chromatography-mass spectrometry data, *Anal. Chem.* **2012**, *84*, 6477-6487.

(3) Koo, I.; Kim, S.; Shi, B.; Lorkiewicz, P.; Song, M.; McClain, C.; Zhang, X., EIder: A compound identification tool for gas chromatography mass spectrometry data, *J. Chromatogr. A* **2016**, *1448*, 107-114.

(4) Samokhin, A. S.; Revel'skii, I. A., Application of principal component analysis to the extraction of pure mass spectra in chemical analysis by gas chromatography/mass spectrometry, *J. Anal. Chem.* **2010**, *65*, 1481-1488.

(5) Stein, S. E., An Integrated Method for Spectrum Extraction and Compound Identification from Gas Chromatography/Mass Spectrometry Data, *J. Am. Soc. Mass Spectrom.* **1999**, *10*, 770-781.

(6) Halket, J. M. K.; Reed, R. I., Analysis of Mixtures .5., *Org. Mass Spectrom.* **1976**, *11*, 881-887.

(7) Meyerson, S., Derivation of Mass Spectra of Individual Compounds from the Spectra of Mixtures, *Anal. Chem.* **1959**, *31*, 174-175.

(8) Satpathy, G.; Tyagi, Y. K.; Gupta, R. K., A Novel Optimised and Validated Method for Analysis of Multi-Residues of Pesticides in Fruits and Vegetables by Microwave-Assisted Extraction (MAE)-Dispersive Solid-Phase Extraction (D-SPE)-Retention Time Locked (RTL)-Gas Chromatography-Mass Spectrometry with Deconvolution Reporting Software (DRS), *Food Chem.* **2011**, *127*, 1300-1308.

(9) Sobcov, H., Analysis of Liquid Hydrocarbon Mixtures by Mass Spectrometry - Application of Matrices and IBM Techniques, *Anal. Chem.* **1952**, *24*, 1386-1388.

(10) You, Y.; Song, L.; Young, M. D.; van der Wielen, M.; Evans-Nguyen, T.; Riedel, J.; Shelley, J. T., Unsupervised Reconstruction of Analyte-Specific Mass Spectra Based on Time-Domain Morphology with a Modified Cross-Correlation Approach, *Anal. Chem.* **2021**, *93*, 5009-5014.

(11) Nishimura, T. In *Fundamentals of Mass Spectrometry*, Hiraoka, K., Ed.; Springer: New York, NY, USA, 2013.

(12) Sleno, L.; Volmer, D., Ion Activation Methods for Tandem Mass Spectrometry, *J. Mass Spectrom.* **2004**, *39*, 1091-1112.

(13) Vekey, K., Internal Energy Effects in Mass Spectrometry, *J. Mass Spectrom.* **1996**, *31*, 445-463.
